# The Impact of Speed-Accuracy Instructions on Spatial Congruency Effects

**DOI:** 10.5334/joc.318

**Published:** 2023-08-23

**Authors:** Herbert Heuer, Peter Wühr

**Affiliations:** 1 Leibniz Research Center for Working Environment and Human Factors (IfADo), DE; 2 TU Dortmund University, DE

**Keywords:** accuracy, speed, SOA, Simon effect, model-based analysis, congruency, compatibility

## Abstract

In many tasks humans can trade speed against accuracy. This variation of strategy has different consequences for congruency effects in different conflict tasks. Recently, Mittelstädt et al. (2022) suggested that these differences are related to the dynamics of congruency effects as assessed by delta plots. With increasing delta plots in the Eriksen flanker task congruency effects were larger under accuracy set, and with decreasing delta plots in the Simon task they were smaller. Here we tested the hypothesis for a single task, making use of the observation that for the Simon task delta plots decline when the irrelevant feature is presented first, but increase when the relevant feature leads. The differences between congruency effects under speed and accuracy instructions confirmed the hypothesized relation to the slope of delta plots. In fact, for similar delta plots in the compared speed-accuracy conditions, the relation should be a straightforward consequence of the shorter and longer reaction times with speed and accuracy set, respectively. However, when relevant and irrelevant features were presented simultaneously, congruency effects were stronger under speed set at all reaction times. For this condition, a supplementary model-based analysis with an extended leaky, competing accumulator model suggested a stronger and longer-lasting influence of the irrelevant stimulus feature. The congruency effects for reaction times were accompanied by congruency effects for error rates when delta plots were decreasing, but not when they were increasing.

## Introduction

The tradeoff between speed and accuracy is a fundamental characteristic of human performance: in most tasks speed can be increased at the costs of accuracy and vice versa. Most extensively this has been studied for the speed and accuracy of movements (Fitts, 1954; Keele, 1968; MacKenzie, 1992) and for the latency and accuracy of simple decisions (Bogacz, Wagenmakers, Forstmann, & Nieuwenhuis, 2009; Heitz, 2014; Pew, 1969; Schouten & Bekker, 1967; Wickelgren, 1977).

Whereas the basic phenomenon of speed-accuracy tradeoff is well established, it is less clear how the set for speed versus accuracy modulates congruency effects as they can be observed in conflict tasks. Recently, Mittelstädt, Miller, Leuthold, Mackenzie, and Ulrich (2022) showed that congruency effects in the Eriksen flanker task (Eriksen, 1995; Eriksen & Eriksen, 1974) and the Simon task (Lu & Proctor, 1995; Simon, 1969) are modulated in opposite directions. Most notably they suggested that this contrast is a consequence of the different dynamics of the two congruency effects. In the flanker task the congruency effect typically increases with increasing reaction times, and under accuracy set – with longer reaction times – it becomes stronger, whereas in the Simon task the congruency effect typically decreases with increasing reaction times, and under accuracy set it becomes smaller.

However, in addition to the dynamics of the congruency effects these conflict tasks differ in other characteristics such as the nature of the conflict involved (cf. Kornblum, 1994) or the different roles of feature-based and spatial selection (Carrasco, 2011) for processing the task-relevant stimulus. The hypothesis of Mittelstädt et al. (2022) presupposes that the opposite modulations of congruency effects across different speed-accuracy strategies do not result from such other differences between the different conflict tasks. Thus it implies that opposite modulations should also arise when the temporal dynamics of congruency effects are varied for one and the same task. Here we test this implication by way of inducing opposite dynamics of the congruency effect in a Simon task.

In a classic version of the Simon task participants respond to the color of a stimulus by pressing a response key with the left or right hand as rapidly and accurately as possible. Thus, color is the task-relevant stimulus feature. Crucially, the colored stimuli are not presented in the center of the display, but in a randomly chosen left or right position. Their position is the task-irrelevant stimulus feature which nevertheless has effects on reaction time and accuracy. In congruent trials, in which the stimulus and the response position are on the same side of a reference point (left/left, right/right), reaction time is shorter (and accuracy is higher) than in incongruent trials, in which stimulus and response positions are on different sides (left/right, right/left).

The dominant account of such congruency effects is in terms of a dual-route model (Kornblum, Hasbroucq, & Osman, 1990). According to this type of model, responses are activated via two routes, one for (intentional) processing of the relevant stimulus feature and the other one for (automatic) processing of the irrelevant feature. To this basic architecture assumptions about the dynamics of processing have been added, specifically the assumption that the influence of the irrelevant feature declines with the passage of time during each reaction-time interval, either passively (Hommel, 1994) or by active suppression (Ridderinkhof, 2002; Van den Wildenberg, Wylie, Forstman, Burle, Hasbroucq, & Ridderinkhof, 2010).

The postulated dynamics of processing the relevant and irrelevant stimulus features have been derived from the observed dynamics of congruency effects. Typically, these are assessed by means of effect functions (e.g. Wiegand & Wascher, 2005) or delta plots (e.g. Van den Wildenberg et al., 2010) which show congruency effects (differences between reaction times in incongruent and congruent conditions) as a function of the mean reaction times of congruent and incongruent conditions for successive bins of the reaction-time distributions (De Jong, Liang, & Lauber, 1994). The variation of congruency effects across increasing reaction times is generally thought to reflect the time course of the influence of irrelevant stimulus features (Proctor, Miles, & Baroni, 2011). Most delta plots are increasing, that is, congruency effects increase with increasing reaction times, for example for the Eriksen flanker task (Burle, Spieser, Servant, & Hasbroucq, 2014; Mittelstädt et al., 2022). Such increase is consistent with the typical increase of reaction-time variability with mean reaction time (Wagenmakers & Brown, 2007). Only for the Simon task with horizontally arranged stimulus and response positions decreasing delta plots have been observed fairly consistently (Burle, Possamai, Vidal, Bonnet, & Hasbroucq, 2002; De Jong et al., 1994; Ellinghaus, Karlbauer, Bausenhart, & Ulrich, 2018; Mittelstädt & Miller, 2020; Mittelstädt et al., 2022; Wiegand & Wascher, 2007). These imply a violation of the typical relation between mean reaction time and reaction-time variability (Zhang & Kornblum, 1997). According to Mittelstädt et al. (2022), the different time courses of congruency effects in the Eriksen and the Simon task form the basis of their different modulations by speed-accuracy strategies.

Here we varied the SOA between the relevant and the irrelevant stimulus feature in a Simon task in addition to instructions which emphasized either speed or accuracy. In a previous study the variation of the SOA resulted in decreasing (SOA ≤ 0; irrelevant feature leading) and increasing (SOA > 0) delta plots (Heuer, Seegelke, & Wühr, 2023). According to the hypothesis of Mittelstädt et al. (2022), Simon effects should be larger for accuracy than for speed instructions with leading relevant stimulus feature (because of increasing delta plots), and they should be smaller with leading irrelevant stimulus feature (because of decreasing delta plots). Such a finding would confirm the relation between the shape of delta plots and the modulation of congruency effects by different speed-accuracy strategies for a single type of task, that is, without a possible confound by processing differences between tasks.

The hypothesized relation between the shapes of delta plots and the effects of speed-accuracy instructions gains a-priori plausibility from the simple fact that reaction times are longer with accuracy instructions than with speed instructions: congruency effects should decrease at longer reaction times when delta plots are decreasing, and they should increase when delta plots are increasing. This is a straightforward relation as long as the dynamics of congruency effects are similar or even identical under both instructions, the only difference being the time interval in which they are effective. However, the different instructions can have additional effects on the processing of relevant and irrelevant stimulus features which could result in differences between slopes or levels of delta plots. For the Simon task such level differences have been documented by Mittelstädt et al. (2022) and by Van Wouwe, Van den Wildenberg, Claassen, Kanoff, Bashore, and Wylie (2014), specifically stronger congruency effects under speed set than under accuracy set across the whole range of reaction times. Such observations suggest that the different effects of speed versus accuracy set on the congruency effects in the Simon task and the Eriksen flanker task could indeed be related to other (or additional) task differences than the different slopes of delta plots.

To obtain insights into the functional consequences of speed and accuracy instructions for the processing of conflicting relevant and irrelevant stimulus features in the two tasks, Mittelstädt et al. (2022) subjected their data to a model-based analysis by means of the Diffusion Model for Conflict Tasks (DMC; Ulrich, Schröter, Leuthold, & Birngruber, 2015). As is mandatory for almost all sequential-sampling models (e.g. Voss, Rothermund, & Voss, 2004; for an exception see Verdonck, Loossens, and Philiastides, 2021), for both tasks response thresholds were lower for speed than for accuracy set. However, other model parameters were also affected. Of particular interest is the finding of a stronger influence of the irrelevant stimulus feature (amplitude of the distractor effect in the DMC model) under speed than accuracy set only in the Simon task, but not in the Eriksen flanker task. For the present data we used an extended Leaky, Competing Accumulator (LCA) model (Heuer et al., 2023) for a model-based analysis which we report in a Supplement. This is a different type of sequential-sampling model than the DMC model, but it has functionally comparable parameters related, for example, to response thresholds and the influence of the irrelevant stimulus feature.

In short, by the hypothesis of Mittelstädt et al. (2022), negative slopes of delta plots, which we expect for leading irrelevant stimulus features, should come along with a smaller congruency effect under accuracy than under speed instructions, whereas positive slopes, which we expect for leading relevant features, should come along with a stronger congruency effect under speed than accuracy instructions. These straightforward predictions can be complicated by additional instruction-dependent variations of delta plots as they have been observed for the Simon task.

## Method

### Participants

Twenty-four students (20 female, 4 male) with mean age of 22.75 years (range 19 – 30 years) participated in the experiment for course credit. All participants were naïve with respect to the purpose of the study and reported normal (or corrected-to-normal) vision. Most of the participants (i.e., 20) were right-handers on self-report. All participants gave their informed consent before the experiment began.

### Apparatus and task

Participants faced a 17-inch monitor in about 50 cm distance in a dimly lit room. Stimulus presentation and response registration was controlled by a program written in ERTS language (Experimental Run Time System; BeriSoft, Frankfurt am Main, Germany). Participants responded to squares presented on the monitor by pressing the left or right control key of a standard keyboard with the index finger of their left or right hand. The mapping of responses to the task-relevant stimulus feature – the red or green color of a square – was balanced across participants. The task-irrelevant feature was the left or right position of the square.

### Design and procedure

Participants came for two sessions separated by, on average, 6 days (range: 4–14 days). In one session they were instructed to respond as quickly as possible, and in the other session they were instructed to respond as accurately as possible. The best three performers under each instruction could win 10 Euro each. The order of the two instruction conditions was balanced across participants.

At the beginning of each session, instructions were presented on the monitor. They described a typical sequence of events in each trial, the stimulus-response mapping, and the speed-accuracy emphasis. In particular, in one session, participants were instructed to respond as quickly as possible, while caring less about errors. In contrast, in the other session, participants were instructed to respond as accurately as possible, while caring less about speed. Participants read the instructions and pressed the space bar to start a practice block of 22 trials. It was followed by ten experimental blocks, each one consisting of 2 warm-up trials (which were not recorded) and 40 experimental trials. The 40 experimental trials were random sequences of two repetitions of all 20 combinations of two stimulus colors, two stimulus locations, and five SOAs.

Each trial started with a blank screen for 500 ms. Thereafter a gray square with a side length of 15 mm (approximately 1.4°) was presented at screen center. It changed its color to red or green 500 ms after its onset and stayed for another 500 ms. Depending on the SOA, the square jumped to a lateral position 80 mm to the left or right of fixation 200 ms before (SOA: –200), 100 ms before (SOA: –100), simultaneously with (SOA: 0), 100 ms after (SOA: +100), or 200 ms after (SOA: +200) its color changed from gray to green or red. Independent of the SOA, in each trial the square was shown (in two colors at two locations) for a total of 1,000 ms.

Response monitoring lasted for 2,000 ms, beginning with the presentation of the gray square at screen center. This allowed us to detect premature responses within a period of 500 ms before the square changed its color to red or green. After each trial an error message (in German) was presented for 2 seconds (a) if participants had pressed a wrong key, (b) if they had responded prematurely or (c) if no response was recorded during the response-monitoring interval. Reaction time was measured from the onset of the first stimulus feature (relevant or irrelevant) until the keypress. Thus, we treat the SOA analogous to an internally generated temporal offset which likely exists even with SOA = 0 because location is a “feature that is notorious for being processed particularly fast” (Cespón, Hommel, Korsch, & Galashan, 2020, p. 1137). This procedural detail differs from other studies in which presentation of the relevant feature, but not the irrelevant one, triggered reaction-time measurements (e.g., Burle et al., 2005; Mackenzie et al., 2022). The only practical consequence of this difference is that reaction times for negative SOAs were longer than at SOA≥0 in the present experiment rather than shorter.

### Data analysis

Each participant performed 40 trials under each of 20 experimental conditions: for five SOAs there were congruent and incongruent trials, with speed and accuracy instructions in different sessions. Of the 19200 trials in total, 42 (0.21%) were outliers with reaction times below 100 ms or no recorded response. For each participant and each condition we computed the error rate, the mean reaction time of correct responses, and five quantiles (.1, .3, .5, .7, .9) of the distributions of reaction times of correct responses. From the quantiles of each pair of congruent and incongruent conditions, we computed the delta plot for each participant, instruction, and SOA.

Delta plots span different ranges of reaction times under speed and accuracy instructions. We compared them in the overlapping range, first, for their overall increase or decrease by the slope of linear regressions (cf. Mackenzie et al., 2022), and second, for their levels by the congruency effects for identical reaction times. These were estimated from third-order polynomials fitted to the delta plots for reaction times at the centers of the reaction-time overlaps of the delta plots under the two instructions.

For the statistical analysis we used ANOVAs and non-parametric tests as described below. For ANOVAs we report partial eta squared ( \[\eta _p^2\]) as effect size and, when appropriate, the Greenhouse-Geisser corrected *p* with the uncorrected degrees of freedom and the correction factor ɛ. In the figures we show the means together with their standard errors. Only in Figure 3 we show the within-participant standard errors which were computed by the procedure of Cousineau (2005) with the correction of Morey (2008).

## Results

We first describe the dynamics of the congruency effect in the various conditions. The variation across SOAs should bear a systematic relation to the difference between the Simon effect under speed and accuracy instructions, which we report subsequently for mean reaction times and error rates.

### Dynamics of the congruency effect

**Figure 1:**
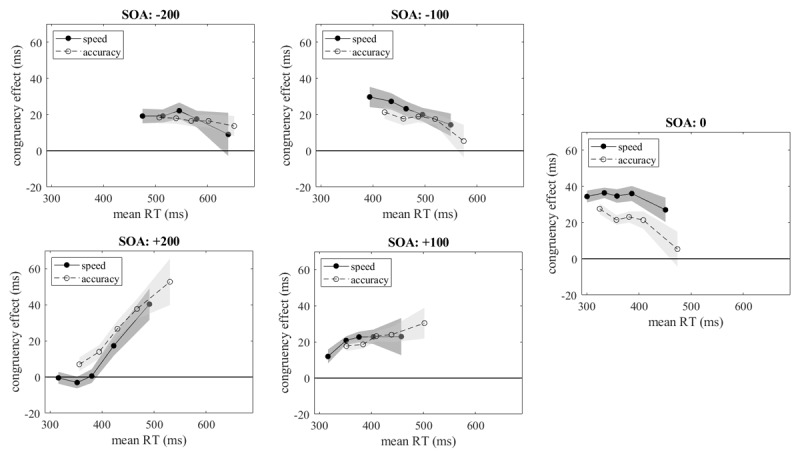
Congruency effects as a function of mean quantiles in congruent and incongruent conditions under speed and accuracy instructions at different SOAs. Shaded areas show the standard errors of the means.

Figure 1 shows the delta plots observed with speed and accuracy instructions at the five SOAs. For SOAs of zero and less (leading irrelevant feature) congruency effects tended to decline as reaction times increased, but with SOAs longer than zero (leading relevant feature) they tended to increase. Under accuracy instructions the delta plots were shifted to the right, reflecting the somewhat longer reaction times in this condition, but in the overlapping range of the two instruction conditions the delta plots were similar with the exception of the zero SOA.

For each participant we computed the slopes of the delta plots across the roughly overlapping parts under both instruction conditions, that is, for quantiles .3, .5, .7, .9 for speed instructions and quantiles .1, .3, .5, .7 for accuracy instructions. These slopes were subjected to an ANOVA with the factors instruction and SOA. Only the main effect of SOA was significant, *F*(4,92) = 16.184, *MSE* = 0.077, *p* < .001, \[\eta _p^2 = {\mathrm{0.413}}\], ɛ = 0.80 (*F* < 1 for both the main effect of instruction and the interaction). The mean slopes [with 95% confidence intervals] were –0.058 [CI: –0.145, 0.029], –0.075 [CI: –0.155, 0.006], –0.066 [CI: –0.155, 0.023], +0.077 [CI: –0.034, 0.189], and +0.302 [CI: 0.238, 0.0.366] for the five SOAs from –200 to +200 ms. Thus, although the slopes were consistently negative for SOA ≤ 0 and positive for SOA > 0, they were not significantly different from zero except at SOA = 200 (with a one-tailed test also at SOA = –100).

For a comparison of the levels of the delta plots under speed and accuracy instruction at each SOA we estimated congruency effects for identical reaction times from third-order polynomials fitted to each delta plot. The identical reaction times used were the centers of the overlapping ranges of reaction times under both instructions (highest mean RT quantile under speed instruction minus shortest mean RT quantile under accuracy instruction): 573, 486, 388, 404, and 424 ms for SOAs of –200, –100, 0, +100, and +200 ms. The differences [with 95% confidence intervals] between the two instructions at these reaction times were 1.9 [CI: –9.7, 13.4], 3.0 [CI: –7.2, 13.2], 12.8 [CI: 2.2, 23.3], 5.7 [CI: –3.7, 15.1]and –14.4 [CI: –33.1, 4.4] ms for the five SOAs. The one-way ANOVA with the factor SOA was significant, *F*(4,92) = 3.021, *MSE* = 790.022, *p* = .039, \[\eta _p^2 = {\mathrm{0.116}}\], ɛ = 0.70. According to the confidence intervals, at SOA = 0 congruency effects were stronger in the speed condition than in the accuracy condition, and at SOA = –200 the difference was numerically reversed, but not significantly different from zero because of high inter-individual variability.

**Figure 2:**
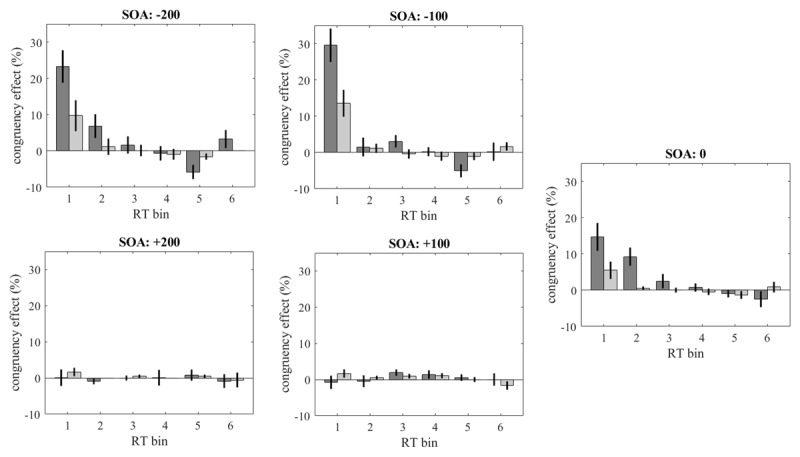
Congruency effects for error percentages at increasing reaction times. The 6 reaction-time bins were bounded by the quantiles [.1 .3 .5 .7 .9] of the distributions of correct reaction times. Congruency effects for speed instruction are shown in dark grey; congruency effects for accuracy instruction are shown in light grey. Vertical lines are standard errors of the means.

The means of the individual congruency effects for error percentages across the six bins defined by the five quantiles of the reaction-time distributions are shown in Figure 2. Congruency effects were primarily found for the fastest 10 or 30% of reaction times, and that only for SOAs ≤ 0. For these SOAs there seemed to be a slight tendency toward reversed congruency effects at long reaction times. With the speed instruction congruency effects were stronger than with the accuracy instruction, but at least a part of this difference is due to the fact that the fastest reaction times in the speed condition were faster than in the accuracy condition. The declining congruency effects for error percentages correspond to the declining congruency effects for reaction times. However, such correspondence does no longer exist for SOAs > 0. Here congruency effects were essentially absent for error percentages, and there was no indication at all that they increased at the longer reaction times.

### Mean reaction times

The individual mean reaction times were subjected to a three-way ANOVA with the factors instruction, congruency, and SOA. All three main effects were statistically significant. Reaction times were faster with speed instruction than with accuracy instruction, 433 vs 463 ms, *F*(1,23) = 28.416, *MSE* = 3800.561, *p* < .001, \[\eta _p^2 = {\mathrm{0.553}}\], they were faster in the congruent than in the incongruent condition, 438 vs 459 ms, *F*(1,23) = 251.968, *MSE* = 200.884, *p* < .001, \[\eta _p^2 = {\mathrm{0.916}}\], and they varied systematically across SOAs (–200 to +200 ms), 563, 481, 380, 402, and 416 ms, *F*(4,92) = 1092.517, *MSE* = 488.155, *p* < .001, \[\eta _p^2 = {\mathrm{0.979}}\], ɛ = 0.56. The difference between instructions was modulated by SOA, giving rise to a significant interaction, *F*(4,92) = 8.862, *MSE* = 231.919, *p* < .001, \[\eta _p^2 = {\mathrm{0.278}}\], ɛ = 0.72: for the three SOAs ≤ 0 the advantage of the speed instruction was 24, 24, and 23 ms, and for SOAs > 0 it was larger, 37 and 43 ms.

**Figure 3:**
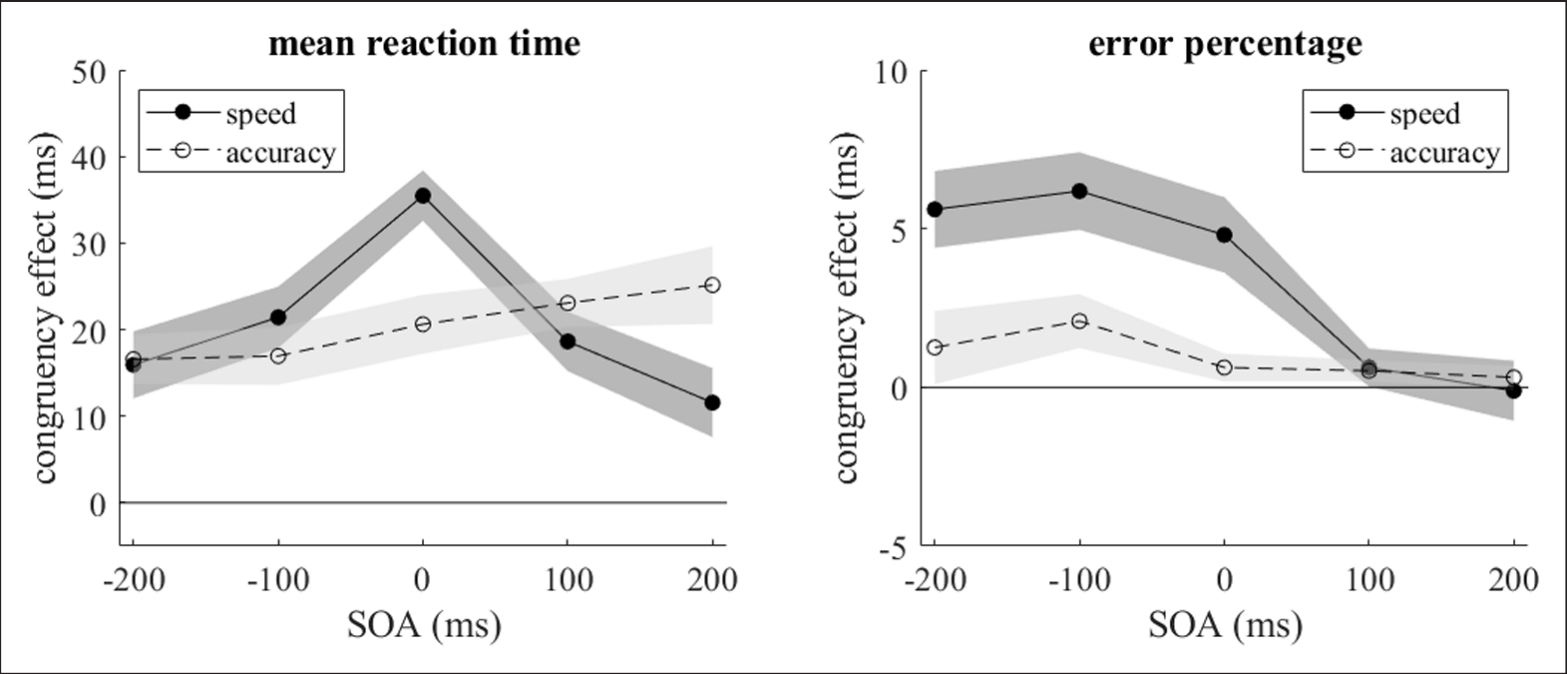
Mean congruency effects (with within-participant standard errors) for individual mean reaction times and error percentages at different SOAs and both instructions (speed and accuracy).

The congruency effect for mean reaction time was modulated by SOA and instruction as shown in Figure 3. Whereas with the accuracy instruction it tended to increase across SOAs from –200 to +200 ms, with the speed instruction it increased to a maximum at SOA = 0 and declined thereafter. Corresponding to the different slopes of delta plots, for SOAs ≤ 0 it was larger than or equal to the congruency effect with the accuracy instruction, but for SOAs > 0 it was smaller. The three-way interaction of instruction, congruency, and SOA was significant, *F*(4,92) = 4.601, *MSE* = 145.235, *p* = .005, \[\eta _p^2 = {\mathrm{0.167}}\], ɛ = 0.79. In addition, the interaction of congruency and SOA was significant, *F*(4,92) = 3.464, *MSE* = 140.981, *p* = .017, \[\eta _p^2 = {\mathrm{0.131}}\], ɛ = 0.82, with congruency effects of 16, 19, 28, 21, and 18 ms at the five SOAs, but not the interaction of instruction and congruency, *F* < 1.

Table 1 presents the slopes of the delta plots at the five SOAs and the differences between the mean congruency effects under accuracy and speed instructions. Overall the data are consistent with the hypothesis of Mittelstädt et al. (2022), but at the SOA of –200 ms the congruency effect should be smaller under accuracy instruction than under speed instruction rather than larger. This deviation is only small and could be chance. At the zero SOA the reduction of the congruency under accuracy instruction is particularly strong, resulting from the lower level of the delta plot with this instruction in addition to its negative slope.

**Table 1:** Mean slopes of delta plots and mean differences between congruency effects under accuracy and speed instructions (negative slopes should be accompanied by negative differences).


SOA	SLOPE	CE DIFFERENCE

–200	–0.058	0.68

–100	–0.075	–4.45

0	–0.066	–14.85

100	0.077	3.87

200	0.302	13.59


### Error percentage

Error percentages were analyzed by non-parametric statistical tests because of frequently skewed distributions. Their means were 7.1, 6.2, 2.9, 1.4, and 1.4% at the five SOAs of –200, –100, 0, +100, and +200 ms, respectively, and for the two instruction conditions they were 5.7% for speed and 1.9% for accuracy. The congruency effects are shown in Figure 3. For both instruction conditions they declined across SOAs, with the decline being significant for the speed condition, χ*^2^*(4) = 33.981, *p* < .001, and almost so for the accuracy condition, χ*^2^*(4) = 8.130, *p* = .087, as indicated by Friedman tests. For the speed condition, congruency effects were larger than zero at SOAs ≤ 0 according to Wilcoxon tests (*T*(24) = 19.5, 26, and 10, all *p* < .001), but for the accuracy condition only at SOA of –100 ms (*T*(24) = 44, *p* = .023). The difference of the congruency effects in the two instruction conditions also declined across SOAs, χ*^2^*(4) = 12.118, *p* < .017. Different from the findings for mean reaction time, however, there was no indication of a reversal of the difference at SOA > 0.

## Discussion

The present experiment had been designed to probe the relation between the slopes of delta plots and the modulation of congruency effects by speed-accuracy tradeoff for a Simon task for which different slopes have been induced by different SOAs between presentations of relevant and irrelevant stimulus features. In addition we found that variations of the SOA had different consequences for reaction-time and accuracy congruency effects. We discuss both these issues in turn.

### Speed-accuracy tradeoff, congruency effects, and the slopes of delta plots

According to a hypothesis proposed by Mittelstädt et al. (2022), decreasing and increasing delta plots, as found in the Simon task and the Eriksen flanker task, respectively, should predict the differences between congruency effects under speed and accuracy sets. Our findings support this hypothesis. Crucially, we did not compare different conflict tasks with different slopes of delta plots but induced such variations in one and the same Simon task. When the task-irrelevant stimulus feature (stimulus position) led the relevant one (stimulus color), slopes of delta plots were negative and Simon effects under accuracy instructions were smaller than (or as small as) under speed instructions. In contrast, when the relevant feature led the irrelevant one, slopes of delta plots were positive and Simon effects under accuracy instructions were larger than under speed instructions. Obviously, these results cannot be attributed to differences between different conflict tasks such as the Eriksen and the Simon task.

The relation between the slopes of delta plots and the differences between congruency effects under speed and accuracy sets could result from the simple fact that responses under accuracy set are slower than under speed set. This would indeed be the main reason for the relation if delta plots were identical under both sets except for covering different ranges of reaction times. With the present data this was the case for SOA ≠ 0, where both the slopes of delta plots and their levels at identical reaction times were not reliably different between speed and accuracy conditions. However, for SOA = 0 we found consistently stronger congruency effects throughout the range of reaction times under speed than under accuracy set. This is not an anomaly of the present data as different levels of delta plots at SOA = 0 have been observed previously (e.g., Van Wouwe et al., 2014; Mittelstädt et al., 2022).

Why are congruency effects under speed set stronger than under accuracy set for the whole range of reaction times (as revealed by the different levels of delta plots) in the Simon task with synchronous presentation of relevant and irrelevant stimulus features, but not with asynchronous presentation or in the Eriksen flanker task? A tentative answer is provided by model-based analyses. Mittelstädt et al (2022), using the Diffusion Model for Conflict Tasks of Ulrich et al. (2015), found for the Simon task, but not for the Eriksen task, an overall stronger influence of the task-irrelevant feature after speed than after accuracy instructions. The present model-based analysis, reported in a Supplement, used an extended version of the Leaky, Competing Accumulator Model (Heuer et al., 2023) and suggested an initially stronger and slower-decaying influence of the task-irrelevant feature after speed than after accuracy instructions only at the zero SOA, but not at the other SOAs. Averaging the model parameters related to processing of the stimulus features across speed and accuracy set produced simulated congruency effects for the zero SOA, but not for the other SOAs, that were too similar to be consistent with the observed ones, confirming the role of differences in stimulus processing. Thus, according to both model-based analyses, in particular under speed set there is a specific difficulty of discarding the influence of the irrelevant stimulus position in the Simon task with (roughly) synchronous presentation of relevant and irrelevant stimulus features.

Identification of the sources of this specific difficulty of attentional selection is beyond the purpose of the present study, but it invites some speculations. Attentional selection can be based on space or stimulus features (e.g. Carrasco, 2011); spatial selection should be involved in discarding the influence of the irrelevant features in the Eriksen flanker task, but feature-based selection in the Simon task. Attentional selection can also be based on time (Nobre & Van Ede, 2018), and this could be part of the preparation for a forthcoming rapid response to a signal (cf. Müller-Gethmann, Ulrich, & Rinkenauer, 2003). At SOA = 0 this top-down selection is combined with bottom-up selection resulting from the abrupt change of the position of the stimulus, and this could enhance the influence of the irrelevant stimulus feature as compared with other SOAs. The simultaneous changes of color and position could also result in stronger binding of these features than temporally separated changes. Such grouping is known to foster congruency effects (e.g. Kramer & Jacobson, 1991); similarly, congruency effects are stronger when irrelevant features belong to the same object as relevant features than when they belong to different objects (Wühr & Waszak, 2003; Wühr, Biebl, Umiltá, & Müsseler, 2009).

### Congruency effects for speed and accuracy

Congruency effects for reaction times are often accompanied by congruency effects for accuracy. In the present experiment the congruency effect for accuracy declined at longer reaction times at all SOA ≤ 0. This decline is more or less in line with the typical overall decline of error rates at longer reaction times, which is also known as micro-speed-accuracy tradeoff (Pachella, 1974). For SOA > 0 the congruency effect on accuracy was small already at short reaction times and remained small at longer ones. The transition from decreasing to increasing congruency effects across the range of SOAs, that was found for reaction times, was absent for error rates. This is consistent with the findings for Simon and Eriksen tasks reported by Mittelstädt et al. (2022, Fig. 2D), where different dynamics of congruency effects were observed only for reaction time, but not for accuracy. Corresponding to their different dynamics, the reversal of the difference between congruency effects under speed and accuracy instructions across the range of SOAs was only seen in the mean reaction times, but not in the error rates.

Dichotomizing what is certainly a continuum, we observed two types of congruency effects. The “early” type is seen in reaction time and accuracy, with strong congruency effects in both measures at short reaction times that decline at longer latencies. The “late” type is seen only in reaction times, with weak congruency effects at short reaction times for both measures that increase at longer latencies only for reaction times, but not for accuracy. In terms of staggered onsets of relevant and irrelevant features, the first type should primarily occur with leading irrelevant features and the second type with leading relevant features. Consistent with this hypothesis, Mackenzie et al. (2022) found reliable congruency effects for error rates in an Eriksen task, a Simon task, and a Stroop task with leading irrelevant features, but with leading relevant features the error-rate congruency effects were reduced for all three tasks and no longer statistically significant for the Eriksen and the Stroop task.

### Conclusions

The present findings suggest three conclusions. First, there is a systematic relation between the dynamics of congruency effects, specifically the slopes of delta plots, and the differences between congruency effects under speed and accuracy set. This relation holds not only across different conflict tasks, but also for variants of the same task. Second, in addition to differences between congruency effects under speed and accuracy instructions that result from the different ranges of reaction times in these conditions, there are differences that result from stronger influences of irrelevant stimulus features under speed instructions. These differences might be restricted to the Simon task with (almost) simultaneous presentation of relevant and irrelevant stimulus features. Third, congruency effects in conflict tasks can be located on a continuum between two extreme types: congruency effects that emerge early in the reaction-time interval show up in reaction times and error rates, but those that emerge only late are restricted to reaction times.

## Data Accessibility Statement

The data reported in this paper has been published on the Mendeley Data Repository (https://data.mendeley.com/datasets/pgv8mxxcgm/1).

## Code availability

The programs, which were used for performing model simulations, can be obtained from the first author upon request.

## Additional File

The additional file for this article can be found as follows:

10.5334/joc.318.s1 Supplement. The supplement describes a model-based analysis of the results reported in the main article.
