# Supplementary material for: The Impact of Speed-Accuracy Instructions on Spatial Congruency Effects
Source: J Cogn. 2023 Aug 23;6(1):49. doi: 10.5334/joc.318 (PMC10453986; doi:10.5334/joc.318)
Supplement: Supplement. — The supplement describes a model-based analysis of the results reported in the main article. [file joc-6-1-318-s1.zip › s1-joc-318_wuhr/Heuer Wuehr sat rev2 supplement clean.docx]

The impact of speed-accuracy instructions on spatial congruency effects**:** Supplement

Popular sequential-sampling models for conflict tasks have been proposed by Hübner, Steinhauser, and Lehle (2010), White, Ratcliff, and Starns (2011), and Ulrich, Schröter, Leuthold, and Birngruber (2015). All these models are extensions of the diffusion model first described by Ratcliff (1978) and developed during the decades since then (e.g., Ratcliff, Smith, Brown, & McKoon, 2016; Voss, Nagler, & Lerche, 2013). The model of Ulrich et al (2015), called the DMC model, has been used by Mittelstädt et al. (2022).

Our analysis is based on an extension of the Leaky, Competing Accumulator (LCA) model first decribed by Usher & McClelland (2001). We have extended this model to take the influence of irrelevant features into account (Wühr & Heuer, 2018), allowing for their declining influence during each trial and a temporal offset between the response activations by relevant and irrelevant features (Heuer et al., 2023). These additions to the basic LCA model are similar to characteristics of a computational model proposed by Zorzi and Umiltá (1995).

**The extended LCA model**

The extended LCA model has two response codes for correct and incorrect responses, respectively. Their instantaneous activations Δa_c_(i) and Δa_e_(i) during each time interval i are defined by two difference equations (cf. Usher & McClelland, 2001, Eq. 3):

(1a) Δa_c_(i) = [ I(i) – λ a_c_(i) – β a_e_(i)] (Δt/τ) + ξ(i) √ (Δt/τ)

(1b) Δa_e_(i) = [ (1-I(i)) – λ a_e_(i) – β a_c_(i)] (Δt/τ) + ξ(i) √ (Δt/τ)

with self-inhibition gain λ, lateral-inhibition gain β, and Gaussian noise ξ(i). The Gaussian noise has zero mean and standard deviation σ_n_. The external inputs I(i) and 1-I(i) add to 1 (cf., Usher & McClelland, 2001, p.559). For I(i) = 0.5 they are thus identical for the correct- and error-response codes, and for I(i) >1 the external input to the error-response code, 1-I(i), is negative so that there is a forward inhibition of the error-response code in addition to the lateral inhibition.

The instantaneous activations of each response code (Eq. 1a, b) are cumulated, beginning at initial values a_c_(0)≥0 and a_e_(0)≥0. One of these initial values can be larger than zero, producing a bias towards the associated response (cf. Wühr & Heuer, 2017). Here we treat the bias as a random variable with a uniform distribution with mean zero and width w_B_, with positive values producing a bias toward the correct response and negative values a bias toward the error response. The total cumulated activation of each code is bounded to be non-negative:

(2a) a_c_(i) = max [ 0, a_c_(i-1) + Δa_c_(i)]

(2b) a_e_(i) = max [ 0, a_e_(i-1) + Δa_e_(i)].

When the activation a_c_(i) or a_e_(i) reaches a threshold *θ*, the respective correct or error response is initiated. A non-decision or residual time *R* with uniform distribution is added to the time needed for the decision. The assumption of a uniform distribution (with mean μ*_R_* and width w*_R_*) is somewhat arbitrary, but common for sequential-sampling models (e.g., Ratcliff & McKoon, 2008), though not undebated (e.g., Verdonck & Tuerlinckx, 2016).

We extended the basic LCA model in two ways. First, we added a declining external input related to the task-irrelevant stimulus feature. More specifically, we defined the total external input I(i) as the sum of two components: the time-invariant contribution I_rel_ of the relevant stimulus feature (relevant input) and the additional time-varying contribution g(t)*ΔI_irr_ of the irrelevant stimulus feature (irrelevant input), with g(t) declining exponentially from 1 to 0 with time constant δ.

The second addition is a variable temporal offset *D* between the relevant and irrelevant input with a uniform distribution of mean μ*_D_* and width w*_D_*. We defined the time at which the relevant input becomes available as t=0; with *d*<0 the irrelevant input leads, and with *d*>0 the relevant input leads. For *d*<0, that is, with leading irrelevant input, the external input is

I(t) = $\left\{ \begin{aligned} 0.5 + g\left( t \right)*\Delta I_{\mathrm{irr}} for t<0 \\ I_{\mathrm{rel}}+ g\left( t \right)*\Delta I_{\mathrm{irr}}\mathrm{for}t\geq0 \end{aligned} \right.$

and for *d*>0, that is, when the relevant input leads, it is

I(t) = $\left\{ \begin{aligned} I_{\mathrm{rel}} for 0\leq t\leq d \\ I_{\mathrm{rel}}+ g\left( t \right)*\Delta I_{\mathrm{irr}}\mathrm{for}t>d \end{aligned} \right.$

with g(t)=$e^{-(t-d)/\delta}$. Table S1 gives an overview of the model parameters.

Table S1: Model parameters (time parameters are in seconds, marked by s)

|  | λ | self-inhibition gain |
| --- | --- | --- |
|  | β | lateral-inhibition gain |
|  | σ_n_ | standard deviation of noise |
|  | I_rel_ | relevant external input |
|  | ΔI_irr_ | additional irrelevant external input |
|  | δ | time constant for decline of irrelevant input (s) |
|  | μ_D_ | mean temporal offset (s) |
|  | w_D_ | width of temporal-offset distribution (s) |
|  | θ | response threshold |
|  | w_B_ | width of response-bias distribution |
|  | μ_R_ | mean residual time (s) |
|  | w_R_ | width of residual-time distribution (s) |

For the simulations we set Δt = 0.001 and τ = 0.1, so that Δt/τ = 0.01. Each cycle i of the simulations comprised two steps. In the first step activations of response codes were updated preliminarily based on the external inputs I(i) and 1-I(i) and the noise ξ(i). In the second step the preliminary updates of the response-code activations were used to apply self-inhibitions and lateral inhibitions to the instantaneous activations which then served to update the response-code activations in cycle i-1 to the activations in cycle i.

For the analysis of the present data we allowed specific parametric differences between the 20 conditions of the experiment. Congruent and incongruent conditions were allowed to differ only in the arithmetic sign of the initial influence of the irrelevant stimulus feature, ΔI_irr_. This is a common simplification (e.g., Heuer et al., 2023; Mittelstädt et al., 2022) that neglects possible differences between the strengths and dynamics of inhibiting and facilitating influences. Regarding the differences between speed and accuracy instructions, sequential-sampling models capture these by different response thresholds, lower for speed and higher for accuracy set (e.g., Voss, Rothermund, & Voss, 2004). However, other processes also seem to be affected by instructions emphasizing speed or accuracy (cf. Mittelstädt et al., 2022). These include the residual time, but also parameters that are related to the processing of the relevant and irrelevant stimulus feature. In fact, modulations of these parameters, which could result from differences of attentional focusing, are of particular interest when it comes to an analysis of the modulation of congruency effects in conflict tasks by speed-accuracy strategies.

**Fitting the model.**

We pooled the data of all participants (neglecting the outliers) for each of the 20 experimental conditions by linear transformations as described by Sternberg (2023). Specifically the linear transformation of reaction time $x_{ij}$ observed in trial *j* of participant *i* in any of the 20 conditions was transformed into $y_{ij}$ as $y_{ij}=m_{.}+\left( x_{ij}-m_{i} \right)\frac{q_{.}}{q_{i}}$ where $m_{i}$ is the mean of participant *i* and $m_{.}$ is the mean of these individual means. Similarly, $q_{i}$ is a robust scale estimator Q_n_ for participant *i* and $q_{.}$ is the mean of the individual scale estimators. The estimator Q_n_ is based on an order statistic of the absolute differences between observations (Rousseeuw & Croux, 1993; Croux & Rousseeuw, 1992). As this procedure of pooling the individual data is not common practice, we computed the absolute deviations between the means of individual estimates and the estimates derived from the pooled data. For the error percentage the mean absolute deviation, computed across the 20 conditions, (in brackets are the maximum deviations) was 0.004% (0.015%; the source of this deviation are different numbers of outliers of the participants), and for mean reaction time it was 0.27 ms (1.15 ms). For the five quantiles (.1, .3, .5, .7, .9) the absolute deviations were 1.22 (4.69), 0.91 (2.17), 0.98 (3.94), 1.59 (4.24), and 4.63 (12.52) ms.

From the pooled data, 954 - 960 trials per condition, we computed the error percentage and 9 quantiles (.1 .2, …, .8, .9) of the reaction-time distribution of correct responses. For each of the five SOAs we estimated the model parameters for congruent and incongruent conditions under speed and accuracy instructions. We estimated the model parameters by minimizing the weighted sum of the squared deviations between predicted and observed relative error frequencies and quantiles of the pooled distributions of reaction times of correct responses. Reaction times of errors were neglected for fitting the model because error frequencies were quite small in some conditions and mean error reaction times extremely unreliable. More specifically, the minimized cost functions were

*C* = $1000 \sqrt{\frac{1}{4*8.75} A}$ with

$A = \sum_{j=1}^{4} \left[ {c_{0} \left( p_{ob.j}-p_{pr.j} \right)}^{2}+\sum_{k=1}^{9} c_{k}\left( P_{kj.ob}-P_{kj.pr} \right)^{2} \right]$,

where *j* = 1, …,4 are the congruent and incongruent conditions under speed and accuracy instructions at one of the five SOAs, *p* is the error probability, *P_k_* are the 9 quantiles of the pooled distributions of reaction times of correct responses, and *c_k_* are the weights (.5, 1, 1, 1, 1, 1, 1, 1, .75,.5 for the error probability and the quantiles in increasing order, 8.75 is the sum of the weights for each of the 4 conditions). Subscripts *ob* and *pr* indicate the observed and predicted data, respectively. Multiplication by 1000 improves readability.

We started each cost minimization with 1000 simulated trials per condition, which were increased up to 100,000 trials, using the MATLAB function *fminsearch*. The initial parameters were set somewhat intuitively to be not too far away from the final estimates with one exception: parameters that could differ for speed and accuracy instructions were set to identical values initially. We used successive runs of the function with 75 iterations. The search ended when with 100,000 trials per condition a criterion was reached that included changes of the parameters and the function value (parameters of *fminsearch* were TolX= 0.3 and TolFun=0.15). (For the SOA of +100 ms the search ended by these criteria although the predicted error percentages were much too small, therefore we re-started the search after manual adjustment to increase error percentages.) After the end of the search, we used the parameter estimates to re-compute the costs and the predictions with another run of 100,000 simulated trials.

Our parameter estimates should be considered with caution for several reasons. First, estimated parameters of simulated data may not recover the parameters that had been used for the simulations (e.g., Hübner & Pelzer, 2020; Miletic et al., 2017; White et al., 2017). Second, different cost functions can produce different results because they place different weights on different aspects of deviations between observed and predicted data. Third, for the minimization of multi-parametric cost functions the risk of ending in local minima is notorious, and there can be “flat regions” in which the costs hardly vary as the parameter space is explored. As a consequence, parameter estimates can vary strongly for almost identical goodness-of-fit criteria (Miletic et al., 2017). Fourth, these problems are aggravated for models like the present one for which there is no closed form of the predicted distribution functions so that they have to be estimated from simulated data. In this case not only the observed data are noisy, but also the predicted data. The search for a minimum of the cost function then is equivalent to finding a minimum on a multidimensional surface that varies its height profile at each glance – keeping that variation sufficiently small requires a large number of simulations, 100,000 in our case for each “glance”. The conclusion from these considerations is that our estimated parameters can account reasonably well for the findings, as we show, but that there may be other sets of parameters that could do so as well. Thus, in addition to model fit, the model parameters should be plausible theoretically and in relation to other similar models.

Taking the estimated parameters for granted, we computed reaction times and error rates for 1000 samples with 960 trials per condition, as in the present experiment, to derive intervals in which 95% of samples with the same size as the one actually collected would fall. These intervals, which we designate as “prediction intervals”, reflect the uncertainty of the model predictions for error rates and reaction times, given the estimated parameters and a sample size corresponding to that of the experiment.

Table S2: Indicators of goodness-of-fit

|  | SOA: -200 ms | SOA: -100 ms | SOA: 0 ms | SOA: +100 ms | SOA: +200 ms |
| --- | --- | --- | --- | --- | --- |
| C | 5.2 | 3.2 | 3.9 | 2.8 | 3.9 |
| χ^2^ | 11.0 | 4.4 | 10.2 | 3.6 | 6.4 |
| $\chi_{e}^{2}$ | 35.8 | 16.5 | 25.1 | 22.2 | 14.6 |

**Results and Discussion**

Table S2 lists the indicators of goodness-of-fit. C is the weighted root mean squared deviation as described above used for fitting the model. From the simulated data additional statistics were computed: χ^2^ is the Chi-squared statistic computed from the observed and predicted frequencies of errors and correct responses in the bins defined by the observed quantiles of the reaction-time distributions of correct responses, and for the computation of $\chi_{e}^{2}$ the error frequency was split into frequencies of errors with reaction times above and below the median. This indicator thus reflects the rather poor prediction of error reaction times which were neglected in fitting the model because of their low reliability.


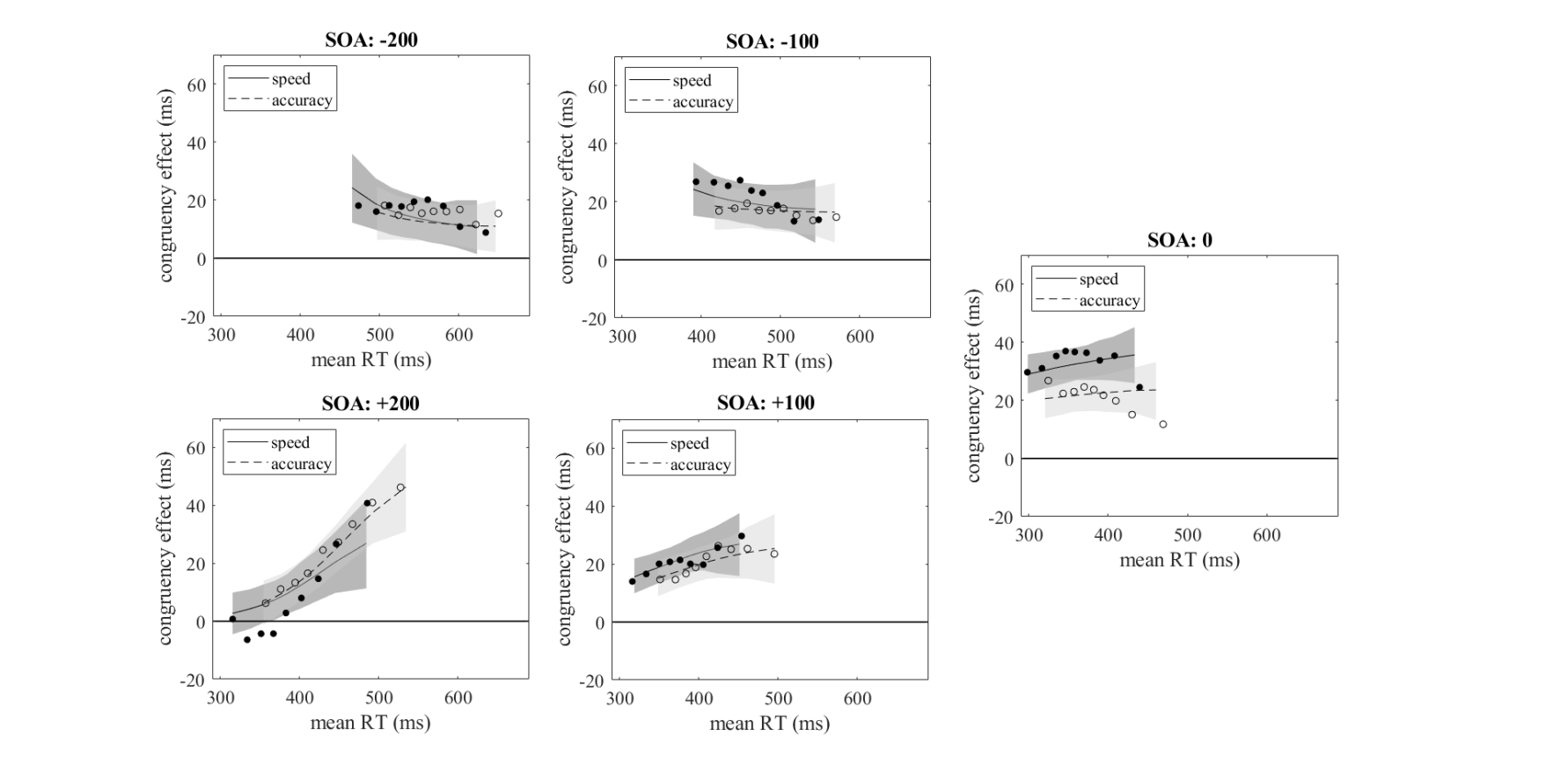


Fig. S1: Continuous and broken lines show the predicted congruency effect as a function of mean quantiles in congruent and incongruent conditions under speed and accuracy instructions at different SOAs. Shaded areas show the 95% prediction intervals. Filled and open circles show the observed congruency effects.

Figure S1 shows the delta plots derived from the estimated model parameters together with the 95% prediction intervals. The observed congruency effects, as estimated from the pooled data (filled and open circles), were within the prediction intervals with only few exceptions. The predicted delta plots were decreasing for negative SOAs and increasing for positive SOAs; only for the SOA of 0 ms the decrease of the observed congruency effects at the longest reaction times was not well captured by the model, but the difference between the delta plots for the two instructions at identical reaction times was.


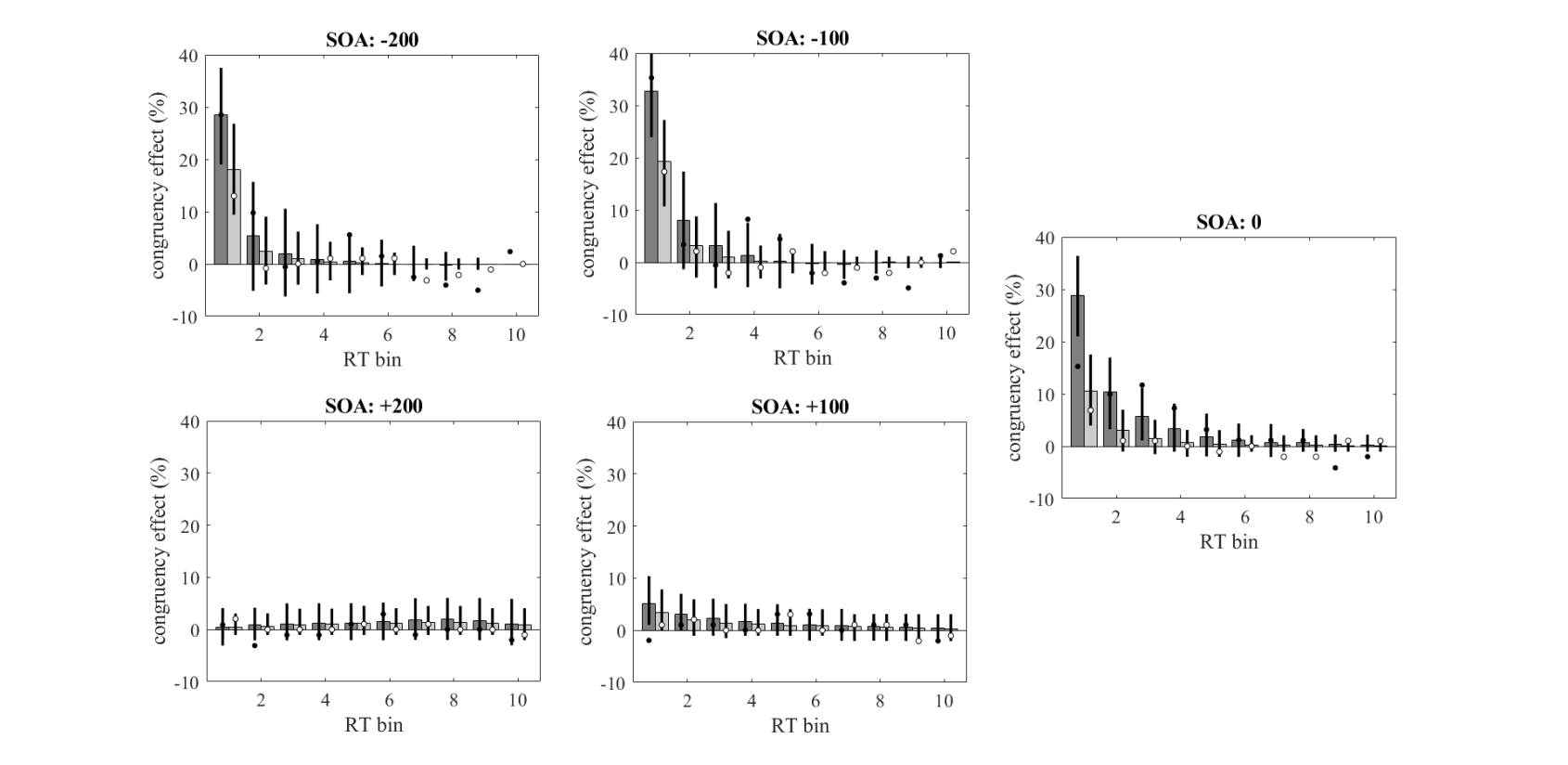


Fig. S2: Predicted congruency effects for error percentages at increasing reaction times. The 10 reaction-time bins were bounded by the quantiles [.1 .2 .3 .4 .5 .6 .7 .8 .9] of the distributions of predicted correct reaction times. Congruency effects for the speed instruction are shown in dark grey; congruency effects for the accuracy instruction are shown in light grey. Vertical bars show the 95% prediction intervals. Filled and open circles show the observed congruency effects for error percentages.

Figure S2 shows the predicted congruency effects for error percentages across increasing bins of the reaction-time distributions together with the observed congruency effects. The variations of the predicted congruency effects across reaction-times are an emergent property of the model; in fitting the model to data only the overall-percentages of errors were used. Consistent with the observed error percentages, substantial congruency effects were primarily found for the fastest reaction times with SOAs≤0, larger for the speed than for the accuracy instruction. Across SOAs, the decline of the congruency effects at the fastest reaction times appeared somewhat slower in the simulated than in the observed data, and the slight negative congruency effects apparent in the observed data at long reaction times at SOAs≤0 were absent in the simulated data. Otherwise, the deviations of the predicted from the observed congruency effects appeared rather piecemeal.


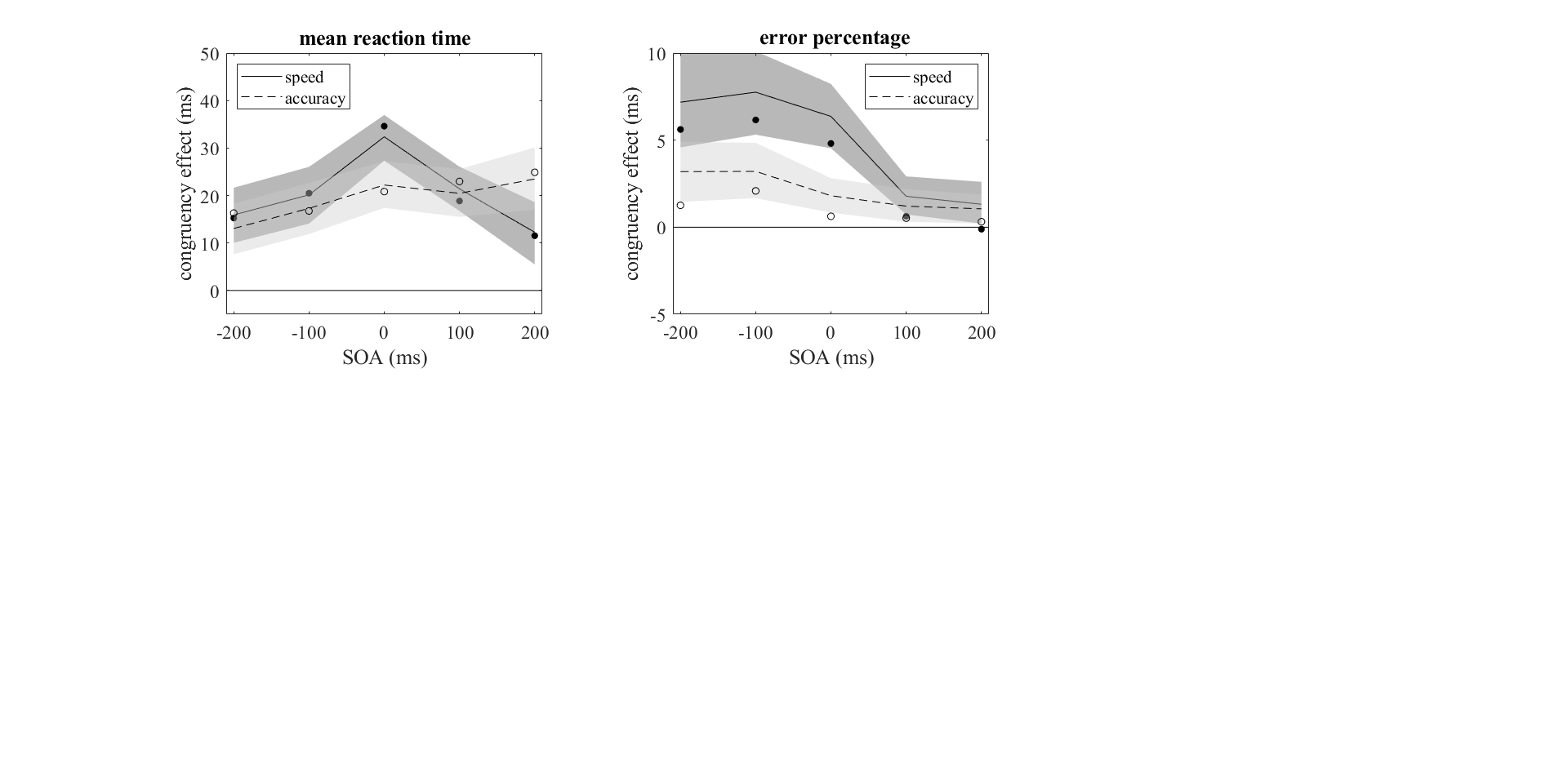


Fig. S3: Predicted congruency effects for mean reaction times and error percentages at different SOAs and both instructions (speed and accuracy). Shaded areas show the 95% prediction intervals. Filled and open circles show the observed means.

Figure S3 shows the predicted congruency effects under speed and accuracy instructions across the five SOAs for mean reaction time and error percentage. For mean reaction time the observed congruency effects were nicely predicted by the model, and the observed means were all in the prediction intervals. For negative SOAs and particularly for the zero SOA the congruency effect was stronger for speed than for accuracy instructions, but at the longest positive SOA, where the increase of the delta plots was strongest, this difference was reversed. For the error percentages the predicted pattern of congruency effects matched the observed pattern fairly well, but the predicted effects were generally too strong, and the observed ones fluctuated around the lower boundaries of the prediction intervals.

Table S3: Observed and predicted (with 95% prediction intervals) mean reaction times of correct responses (mRTc), error percentages (EP), and mean reaction times of errors (mRTe). Observed statistics are from pooled individual data.

|  |  |  | mRTc | | EP | | mRTe | |
| --- | --- | --- | --- | --- | --- | --- | --- | --- |
|  |  |  | obs | pre | obs | pre | obs | pre |
| -200 | speed | con | **543** | *533* 537 *541* | 7.8 | *5.0* 6.5 *8.0* | **517** | *447* 461 *475* |
|  |  | inc | **559** | *549* 553 *557* | 13.4 | *11.7* 13.8 *15.7* | **485** | *438* 449 *458* |
|  | accur | con | 567 | *561* 565 *569* | 3.0 | *1.5* 2.5 *3.3* | **520** | *456* 477 *499* |
|  |  | inc | **583** | *574* 578 *582* | 4.3 | *4.1* 5.6 *7.0* | **503** | *452* 467 *480* |
| -100 | speed | con | 458 | *452* 456 *460* | 5.9 | *3.3* 4.7 *5.9* | **436** | *377* 390 *405* |
|  |  | inc | 479 | *472* 476 *480* | 12.0 | *10.4* 12.4 *14.4* | **400** | *372* 380 *388* |
|  | accur | con | 485 | *479* 483 *486* | 2.4 | *0.9* 1.7 *2.7* | **440** | *383* 406 *428* |
|  |  | inc | 502 | *496* 500 *504* | 4.5 | *3.7* 4.8 *6.3* | **431** | *382* 396 *409* |
| 0 | speed | con | 351 | *344* 348 *351* | **2.1** | *0.4* 0.9 *1.5* | **371** | *290* 322 *353* |
|  |  | inc | **385** | *377* 380 *384* | 6.9 | *5.6* 7.3 *9.0* | **337** | *299* 309 *320* |
|  | accur | con | **381** | *373* 377 *380* | 0.9 | *0.1* 0.4 *0.9* | **402** | *290* 339 *388* |
|  |  | inc | 402 | *395* 399 *403* | 1.6 | *1.4* 2.3 *3.2* | **378** | *308* 325 *345* |
| +100 | speed | con | 374 | *368* 371 *374* | **1.9** | *0.1* 0.5 *1.0* | 344 | *316* 370 *426* |
|  |  | inc | 392 | *389* 393 *396* | 2.5 | *1.4* 2.3 *3.3* | 382 | *341* 360 *384* |
|  | accur | con | 409 | *404* 408 *412* | 0.3 | *0.1* 0.5 *0.9* | **557** | *359* 408 *480* |
|  |  | inc | 432 | *424* 428 *432* | **0.8** | *0.9* 1.6 *2.6* | 396 | *371* 401  *432* |
| +200 | speed | con | 389 | *384* 388 *392* | **2.4** | *0.6* 1.3 *1.9* | 369 | *361* 403 *454* |
|  |  | inc | 401 | *396* 400 *405* | 2.3 | *1.6* 2.6 *3.5* | 390 | *385* 415 *447* |
|  | accur | con | 425 | *423* 427 *431* | 0.3 | *0.0* 0.4 *0.8* | 526 | *359* 436 *556* |
|  |  | inc | 450 | *445* 450 *455* | **0.6** | *0.7* 1.4 *2.3* | 452 | *420* 458 *507* |

Table S3 lists for all 20 conditions the observed and predicted mean reaction times of correct responses, error percentages, and mean reaction times of errors. Whereas the predicted values are from simulations with 100,000 trials per condition, from which the goodness-of-fit indicators of Table S2 were computed, the 95% prediction intervals for the means, in cursive script in Table S3, were derived from 1000 simulations of the 960 trials for each of the 20 conditions, that is, with a similar uncertainty as the observed data. Observed statistics outside the prediction intervals are in bold. For mean reaction times such observations were in 5 of the 20 conditions, and the deviations amounted to only one or two milliseconds. For error percentages again 5 such cases were present, with deviations up to 0.9 %. Thus, model predictions seem reasonably accurate.

Predictions of the error reaction times were obviously poor. However, although the model generally predicts reaction times of errors incorrectly, the differences between mean reaction times of correct and error responses bear a systematic relation to the observed differences across the 20 experimental conditions, as illustrated in Figure S4.


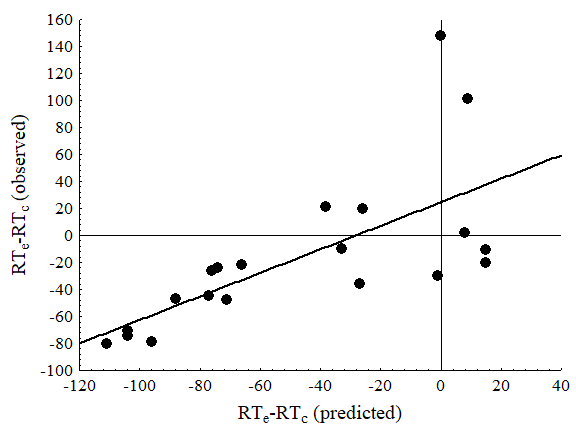


Fig. S4: Regression of the difference between observed mean reaction times of errors and correct responses on the difference between predicted means across the 20 conditions of the experiment.

The deviations of the observed error reaction times from the predicted ones were always of the same kind: they were longer than the range of predicted reaction times. This discrepancy between model and human performance could indicate failures of human performance that are known as lapses and that are most conspicuous in sleep-deprivation research (Dinges & Kribbs, 1991; Tilley & Brown, 1992). However, they are present in essentially all tasks requiring sustained attention (cf. Esterman & Rothlein, 2019). Functionally they can be characterized as temporary goal neglect (cf. Duncan, Emslie, Williams, Johnson, & Freer, 1996), and neurally they are distinguished by reduced frontal activity before stimulus presentation and reduced stimulus-contingent activity changes thereafter (Weissman, Roberts, Visscher, & Woldorff, 2006). Such contaminations of normal processing by lapsing lead to late more or less chance responses at long reaction times, which have comparatively little influence on mean reaction times of many correct responses, but a stronger influence on mean reaction times of only few error responses.

The estimated model parameters are presented in Table S4. Differences between congruent and incongruent conditions were constrained to the arithmetic sign of the initial irrelevant input (ΔI_irr_). Differences between speed and accuracy instructions, however, were allowed to appear in five parameters. In fitting the model, each of these parameters was initially set to identical values for both instruction conditions. Consistent with what one would expect for sequential-sampling models, the estimated thresholds θ were higher under accuracy than under speed instructions at all SOAs. Similarly, at all SOAs the estimated mean residual time μ_R_ was longer with accuracy than with speed instruction. In contrast to these two parameters, the three parameters related to the effects of the relevant and irrelevant input featured no instruction-related differences that were invariant across the five SOAs. For the relevant input (I_rel_) the variations appeared random: they were small and with no systematic relation to the SOA. That was somewhat different for the irrelevant input. In particular, at SOAs of 0 and +100 ms the initial influence of the irrelevant input (ΔI_irr_) was stronger with the speed than with the accuracy instruction, and at SOA of +200 this difference was reversed. The time constant of the decline of the influence of the irrelevant input (δ) was similar for both instructions at all SOAs except for the SOA of 0 ms where the time constant was longer for the speed instruction. Thus, with the zero SOA the initial influence of the irrelevant stimulus feature was stronger and its decline slower under speed than under accuracy set, giving rise to the difference between delta plots at this SOA.

Table S4: parameter estimates

|  | SOA: -200 ms | | SOA: -100 ms | | SOA: 0 ms | | SOA: +100 ms | | SOA: +200 ms | |
| --- | --- | --- | --- | --- | --- | --- | --- | --- | --- | --- |
|  | speed | accur | speed | accur | speed | accur | speed | accur | speed | accur |
| λ | 0.262 | | 0.204 | | 0.278 | | 0.262 | | 0.280 | |
| β | 0.212 | | 0.210 | | 0.216 | | 0.229 | | 0.255 | |
| σ_n_ | 0.216 | | 0.322 | | 0.292 | | 0.335 | | 0.368 | |
| I_rel_ | 1.210 | 1.239 | 1.209 | 1.254 | 1.021 | 1.039 | 0.984 | 0.942 | 0.875 | 0.898 |
| ΔI_irr_ | ±0.142 | ±0.141 | ±0.179 | ±0.169 | ±0.300 | ±0.260 | ±0.262 | ±0.217 | ±0.177 | ±0.265 |
| δ | 0.052 | 0.050 | 0.067 | 0.064 | 0.058 | 0.044 | 0.042 | 0.043 | 0.045 | 0.048 |
| μ_D_ | -0.100 | | -0.088 | | -0.085 | | -0.110 | | -0.107 | |
| w_D_ | 0.115 | | 0.100 | | 0.099 | | 0.142 | | 0.113 | |
| θ | 1.274 | 1.410 | 1.357 | 1.533 | 1.045 | 1.163 | 1.019 | 1.095 | 1.053 | 1.172 |
| w_B_ | 0.312 | | 0.356 | | 0.394 | | 0.446 | | 0.528 | |
| μ_R_ | 0.198 | 0.203 | 0.212 | 0.217 | 0.196 | 0.204 | 0.253 | 0.270 | 0.252 | 0.278 |
| w_R_ | 0.104 | | 0.094 | | 0.087 | | 0.082 | | 0.095 | |

To assess the impact of the parametric differences between speed and accuracy instructions related to the processing of relevant and irrelevant stimulus features (I_rel_ , ΔI_irr_, δ), we simulated the model after averaging the parameters for the two instructions. The resulting delta plots are shown in Figure S5. The differences to the delta plots of Figure S1 are remarkably small. The only conspicuous difference is present at SOA=0, where also the number of observed congruency effects outside the 95% prediction intervals is larger in Figure S5 than in Figure S1. A less conspicuous difference to the delta plots of Figure S1 is apparent at SOA=+200 where under speed instructions congruency effects were essentially absent at the short reaction times, giving rise to a comparatively small parameter ΔI_irr_ as compared with the accuracy instruction (see Table S4).


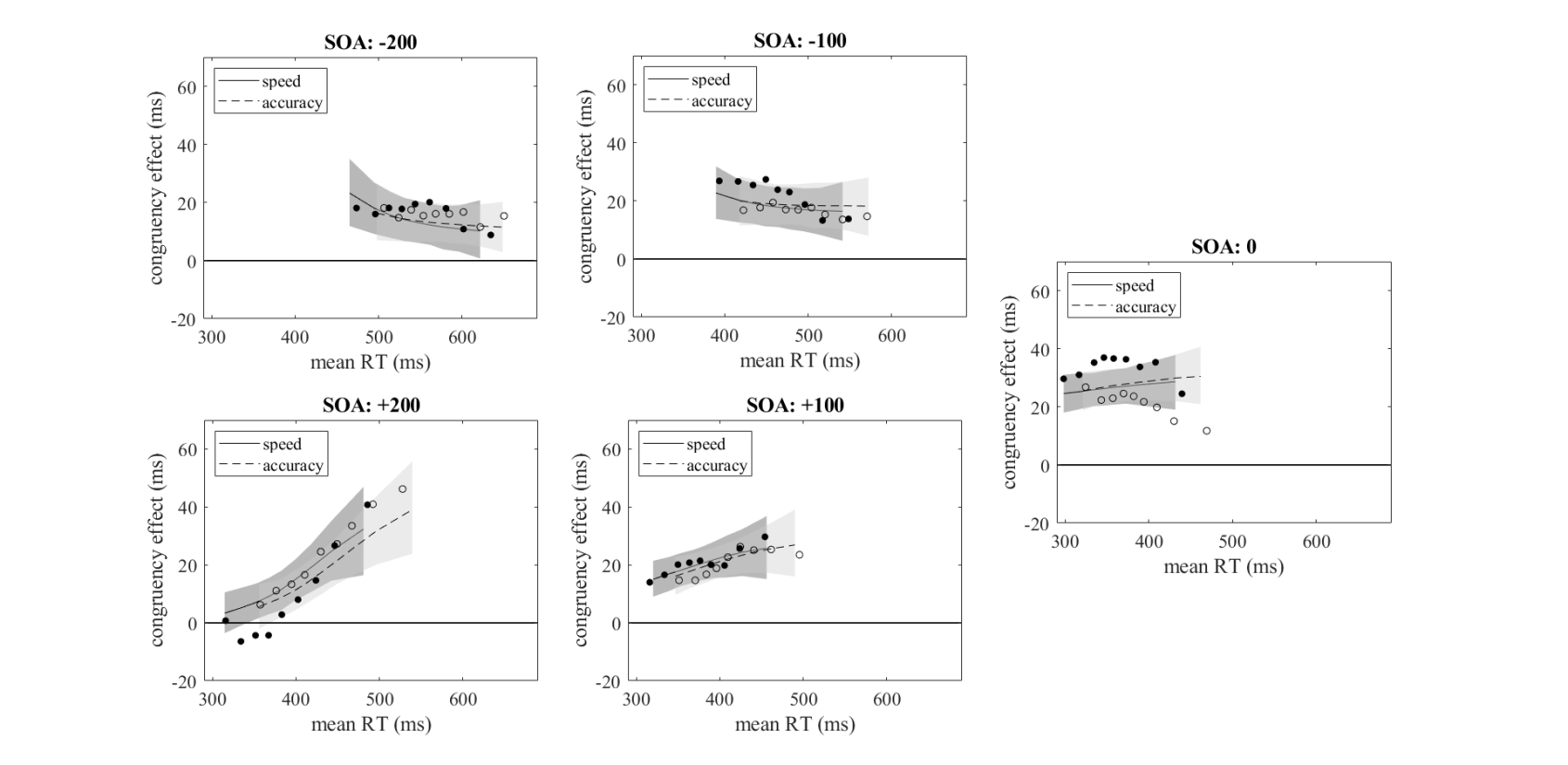


Fig. S5: Simulations with identical parameters I_rel_ , ΔI_irr_, δ for speed and accuracy instructions. Continuous and broken lines show the congruency effect as a function of mean quantiles in congruent and incongruent conditions under speed and accuracy instructions at different SOAs. Shaded areas show the 95% prediction intervals, filled and open circles the observed congruency effects.

Congruency effects for mean reaction times and error rates as predicted by the model after removing the parametric differences related to processing of the relevant and irrelevant stimulus features are shown in Figure S6. As compared with the simulation results shown in Figure S3, the strongest effect of the absent parametric differences is at SOA=0. Here the observed congruency effects under speed and accuracy instructions were no longer within their respective prediction intervals. Their difference was too large to be consistent with identical stimulus processing under speed and accuracy set. This observation, as well as the effect of removing the parametric differences on the delta plots, supports the conclusion that with simultaneous presentation of relevant and irrelevant stimulus features the impact of the irrelevant stimulus feature is enhanced under speed set as compared with accuracy set. For SOA=200 the effects of reducing the parametric differences were weaker, and for the error percentages they were rather marginal at all SOAs.


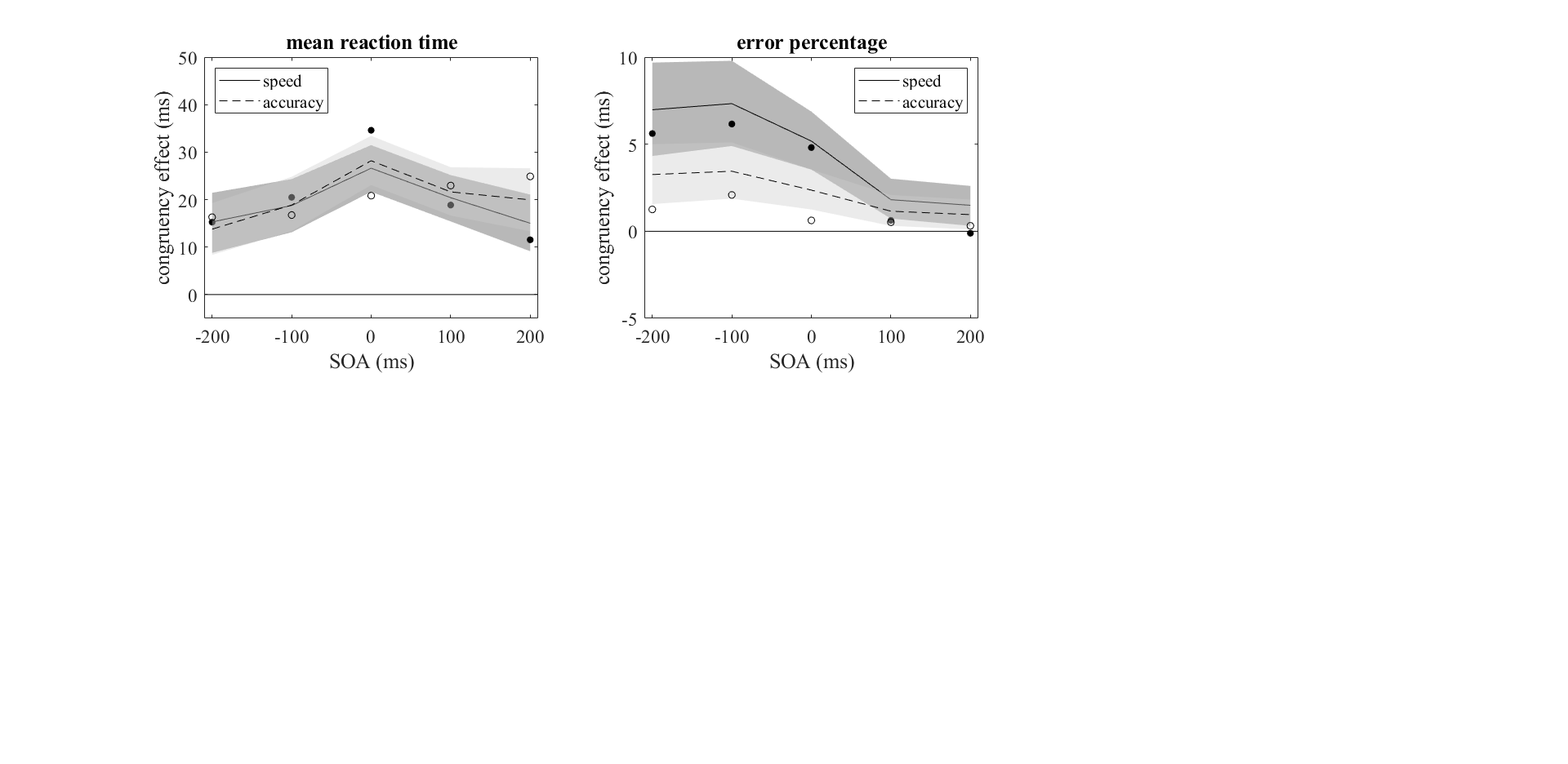


Fig. S6: Simulations with identical parameters I_rel_ , ΔI_irr_, δ for speed and accuracy instructions. Predicted congruency effects for mean reaction times and error percentages at different SOAs and both instructions (speed and accuracy). Shaded areas show the 95% prediction intervals, filled and open circles the observed means.

Regarding the parameter variations across SOAs, some of them appear systematic. With negative SOAs, the influence of the relevant stimulus feature (I_rel_) was stronger than with positive SOAs, the initial influence of the irrelevant stimulus feature (ΔI_irr_ ) was weaker, the time constant of its decline (δ) was longer, and the response threshold (θ ) was higher. Most of these variations can be related to the initial activation of response codes by only the irrelevant input when SOAs are negative. This could lead to a high error rate, which likely was counteracted by keeping the initial influence of the irrelevant input small and by raising the response threshold, and by giving a strong influence – above 1 - to the relevant input as soon as it becomes available, which effectively amounts to a forward inhibition of the error-response code.

A conspicuous difference of almost 60 ms was observed for the mean residual time (μ_R_) between SOAs less or equal zero (205 ms) and positive SOAs (263 ms). This difference is likely related to the fact that with positive SOAs the relevant stimulus feature color was presented first, whereas with the other SOAs the irrelevant feature position was presented first or simultaneously with color: depending on which stimulus feature was presented first, the time needed for early sensory processing could have differed, being shorter for the change of position than for the change of color. In addition to the longer residual time with positive SOAs, the difference between speed and accuracy conditions was increased. This increasing difference across SOAs – 5, 5, 8, 17, and 26 ms – matches the increase of the difference between observed mean reaction times in the two instruction conditions – 24, 24, 23, 37, and 43 ms. Subtracting the differences between estimated residual times from the observed differences between mean reaction times leaves 19, 19, 17, 20, and 17 ms, thus a fairly constant estimate of 18 ms as speed-up under speed instruction due to threshold adjustments and modulations of response-code activations.

**References**

Croux, C. & Rousseeuw, P. J. (1992) Time-efficient algorithms for two highly robust estimators of scale. In: Y. Dodge and J. Whittaker (eds.). Computational Statistics, Volume 1. (pp. 411-428). Heidelberg: Physika-Verlag.

Dinges, D. F. & Kribbs, N. B. (1991). Performing while sleepy: effects of experimentally-induced sleepiness. In: T. H. Monk (Ed.), Sleep, sleepiness and performance. (pp. 97‑128) Chichester: Wiley.

Duncan, J., Emslie, H., Williams, P., Johnson, R., & Freer, C. (1996). Intelligence and the frontal lobe: the organization of goal-directed behavior. Cognitive Psychology, 30, 257‑303.

Esterman, M. & Rothlein, D. (2019). Models of sustained attention. Current Opinion in Psychology, 29, 174-180.

Heuer, H., Seegelke, C., & Wühr, P. (2023). Staggered onsets of processing relevant and irrelevant stimulus features produce different dynamics of congruency effects. Journal of Cognition, 2023, 6: 8. DOI: https://doi.org/10.5334/joc.252

Hübner, R. & Pelzer, T. (2020). Improving parameter recovery for conflict drift-diffusion models. Behavior Research Methods, 52, 1848–1866. https://doi.org/10.3758/s13428-020-01366-8

Hübner, R., Steinhauser, M., & Lehle, C. (2010). A dual-stage two-phase model of selective attention. Psychological Review, 117, 759-784.

Miletić, S., Turner, B. M., Forstmann, B. U., & van Maanen, L. (2017). Parameter recovery for the leaky competing accumulator model. Journal of Mathematical Psychology, 76, 25-50.

Mittelstädt, V., Miller, J., Leuthold, H., Mackenzie, I. G., & Ulrich, R. (2022). The time-course of distractor-based activation modulates effects of speed-accuracy tradeoffs in conflict tasks. Psychonomic Bulletin & Review, 29, 837–854. https://doi.org/10.3758/s13423-021-02003-x

Ratcliff, R. (1978). A theory of memory retrieval. Psychological Review, 85, 59‑108.

Ratcliff, R. & McKoon, G. (2008). The diffusion decision model: Theory and data for two-choice decision tasks. Neural Computation, 20, 873-922.

Ratcliff, R., Smith, P. L., Brown, S. D., & McKoon, G. (2016). Diffusion decision model: current issues and history. Trends in Cognitive Sciences, 20, 260-281.

Rousseeuw, P. J., & Croux, C. (1993). Alternatives to the median absolute deviation. Journal of the American Statistical Association, 88(424), 1273-1283.

Sternberg, S. (2023). Combining reaction-time distributions to conserve shape. Behavior Research Methods, https://doi.org/10.3758/s13428-023-02084-7

Tilley, A. & Brown, S. (1992). Sleep deprivation. In: A. P. Smith & D. M. Jones (Eds.), Handbook of human performance. Vol. 3: State and trait. (pp. 237‑259) London: Academic Press.

Ulrich, R., Schröter, H., Leuthold, H., & Birngruber, T. (2015). Automatic and controlled stimulus processing in conflict tasks: superimposed diffusion processes and delta functions. Cognitive Psychology, 78, 148-174.

Usher, M. & McClelland, J. L. (2001). The time course of perceptual choice: The leaky, competing accumulator model. Psychological Review, 108, 550‑592.

Verdonck, S. & Tuerlinckx, F. (2016). Factoring out nondecision time in choice reaction time data: Theory and implications. Psychological Review, 123, 208-218.

Voss, A., Nagler, M., & Lerche, V. (2013). Diffusion models in experimental psychology. Experimental Psychology, 60, 385-402.

Voss, A., Rothermund, K., & Voss, J. (2004). Interpreting the parameters of the diffusion model: An empirical validation. Memory & Cognition, 32, 1206-1220.

Weissman, D. H., Roberts, K. C., Visscher, K. M., & Woldorff, M. G. (2006). The neural bases of momentary lapses in attention. Nature Neuroscience, 9, 971-978.

White, C.N., Ratcliff, R., & Starns, J.J. (2011). Diffusion models of the flanker task: Discrete versus gradual attentional selection. Cognitive Psychology, 63, 210-238.

White, C. N., Servant, M., & Logan, G. D. (2017). Testing the validity of conflict drift-diffusion models for use in estimating cognitive processes: A parameter-recovery study. Psychonomic Bulletin & Review, 25, 286-301.

Wühr, P. & Heuer, H. (2017). Response preparation, response conflict, and the effects of irrelevant flanker stimuli. Advances in Cognitive Psychology, 13, 70-82.

Wühr, P. & Heuer, H. (2018). The impact of anatomical and spatial distance between responses on response conflict. Memory & Cognition, 46, 994-1009.

Zorzi, M. & Umiltá, C. (1995). A computational model of the Simon effect. Psychological Research, 58, 193‑205.
