# Supplementary figures and images for: The Impact of Speed-Accuracy Instructions on Spatial Congruency Effects
Source: J Cogn. 2023 Aug 23;6(1):49. doi: 10.5334/joc.318 (PMC10453986; doi:10.5334/joc.318)

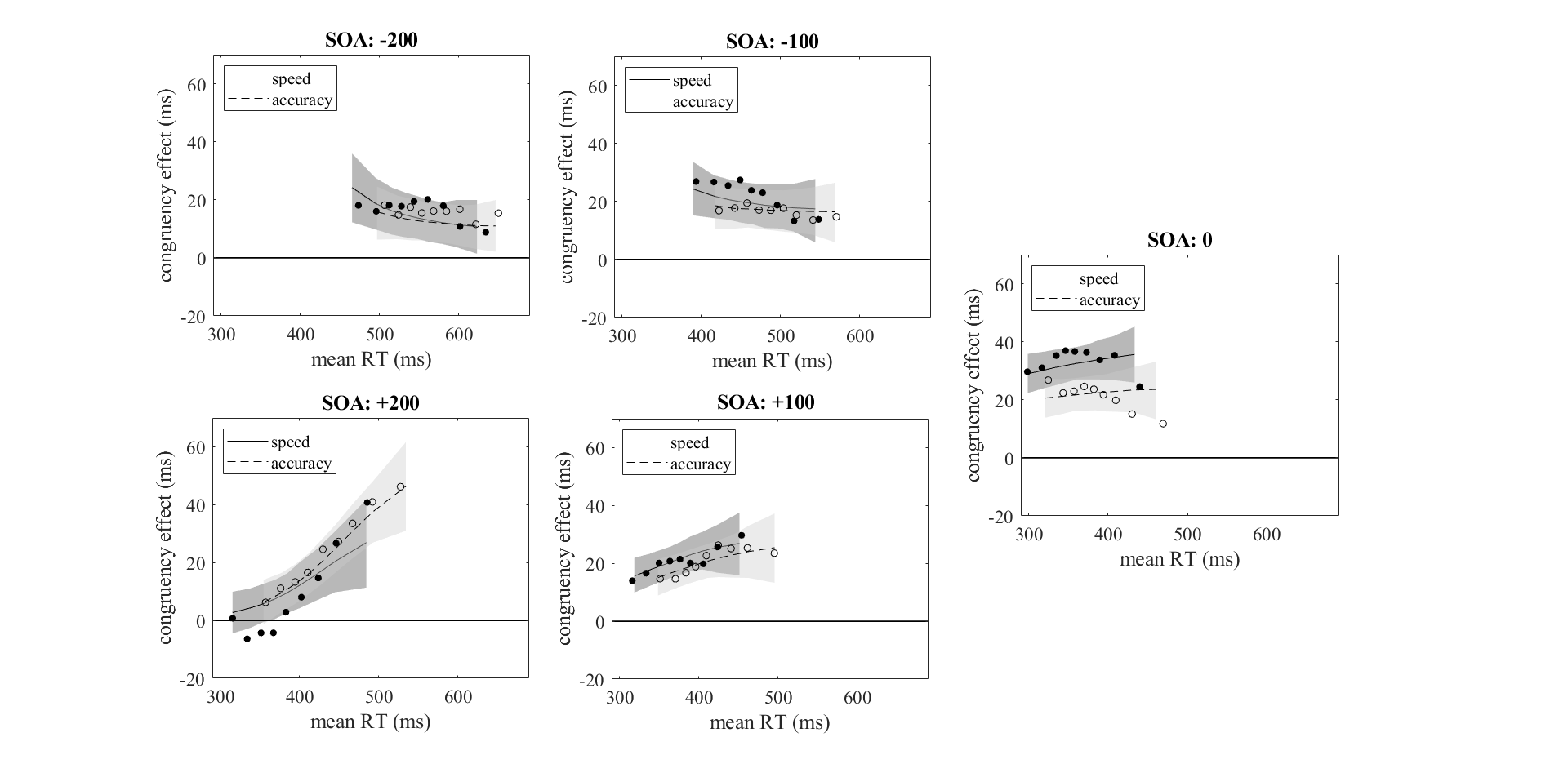

Supplement: Supplement. — The supplement describes a model-based analysis of the results reported in the main article. [file joc-6-1-318-s1.zip › s1-joc-318_wuhr/Fig. S1.tif]

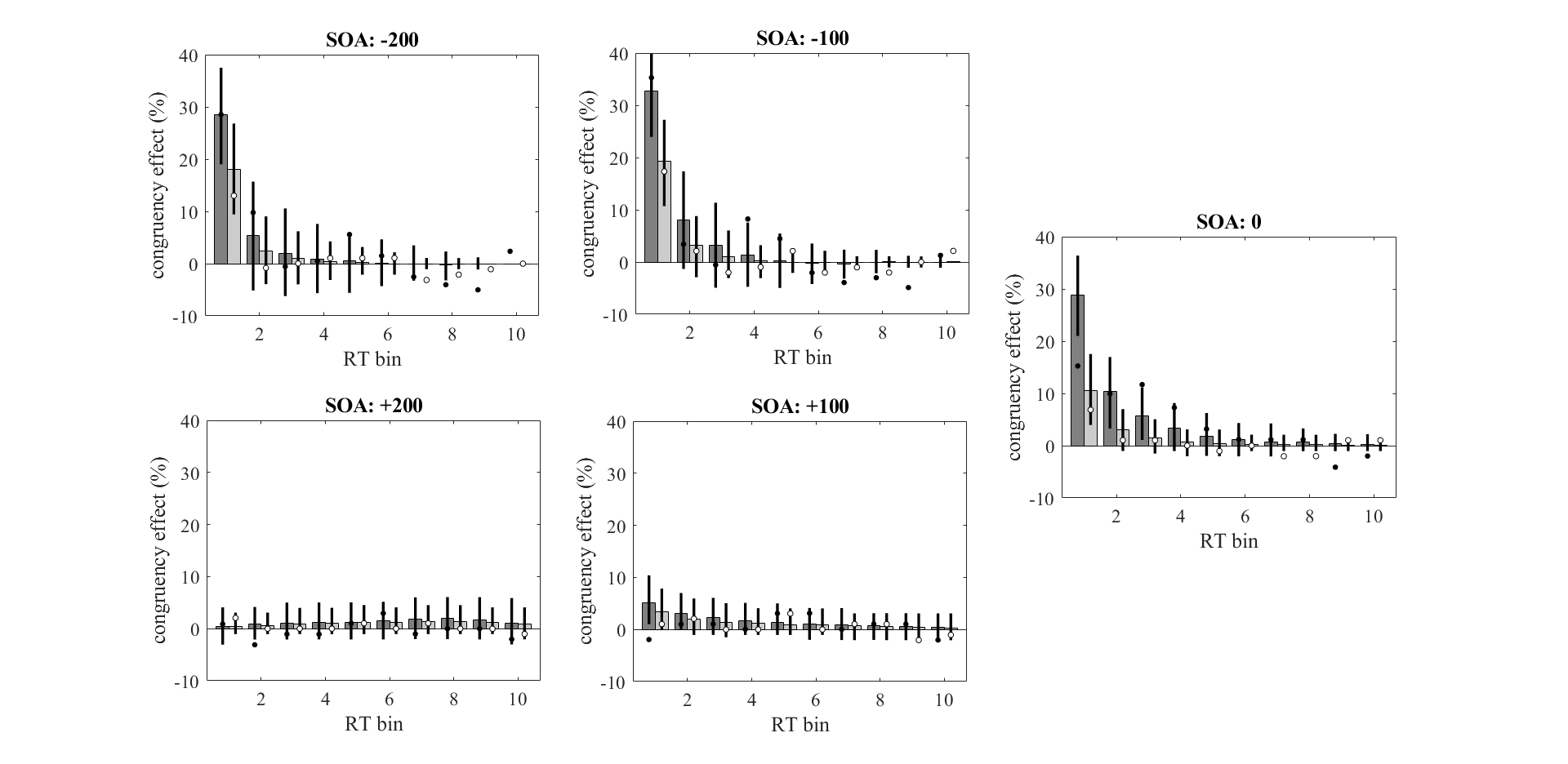

Supplement: Supplement. — The supplement describes a model-based analysis of the results reported in the main article. [file joc-6-1-318-s1.zip › s1-joc-318_wuhr/Fig. S2.tif]

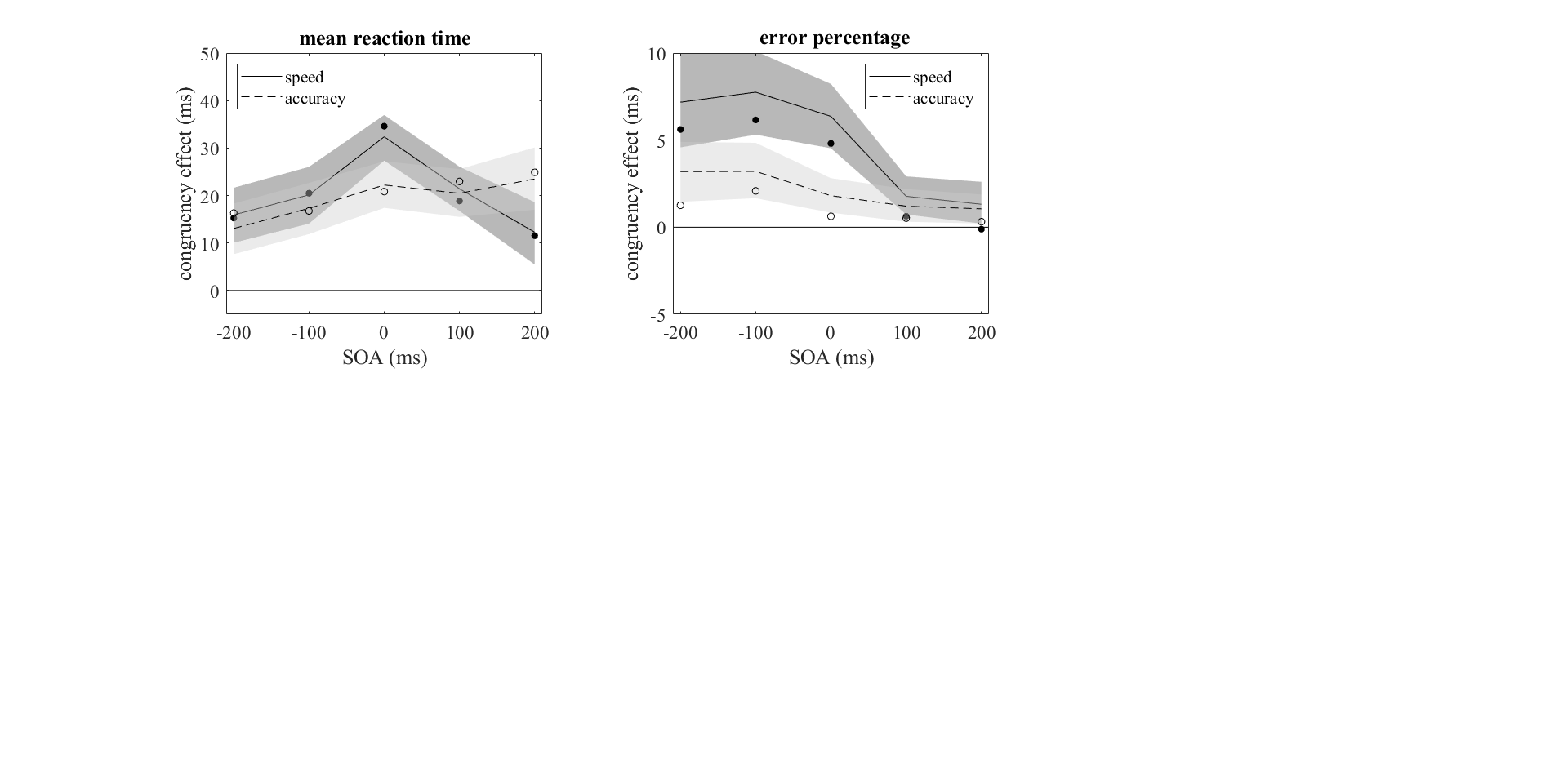

Supplement: Supplement. — The supplement describes a model-based analysis of the results reported in the main article. [file joc-6-1-318-s1.zip › s1-joc-318_wuhr/Fig. S3.tif]

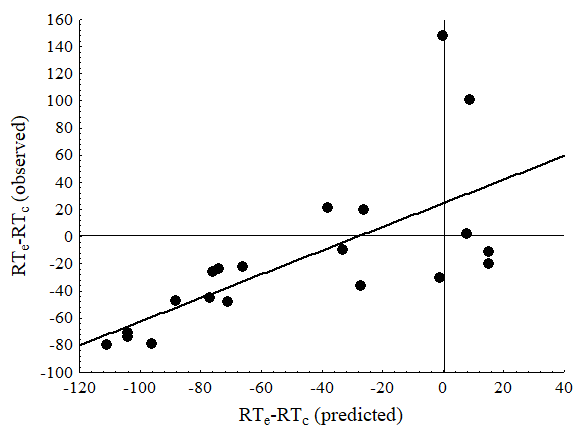

Supplement: Supplement. — The supplement describes a model-based analysis of the results reported in the main article. [file joc-6-1-318-s1.zip › s1-joc-318_wuhr/Fig. S4.tif]

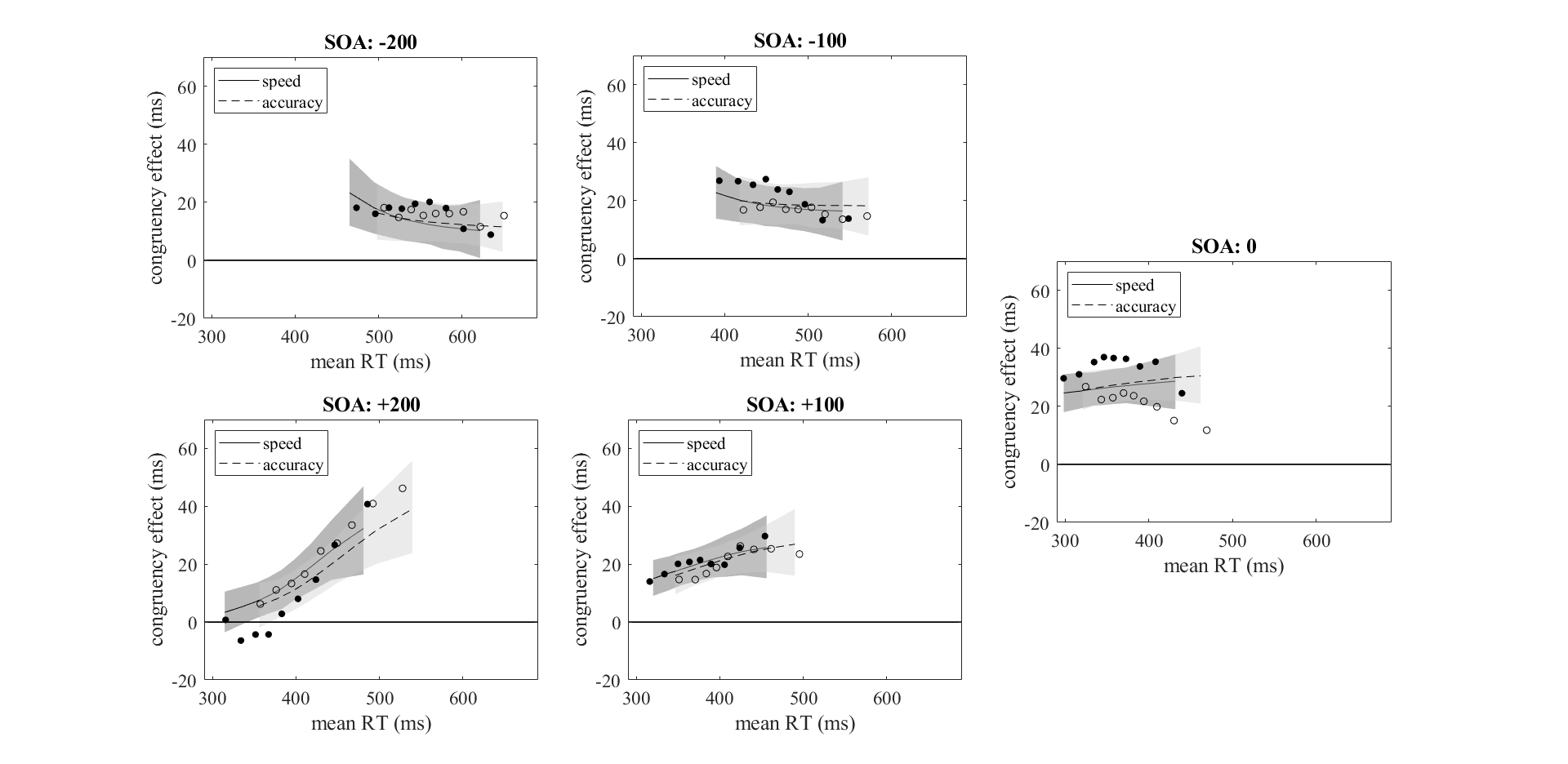

Supplement: Supplement. — The supplement describes a model-based analysis of the results reported in the main article. [file joc-6-1-318-s1.zip › s1-joc-318_wuhr/Fig. S5.tif]

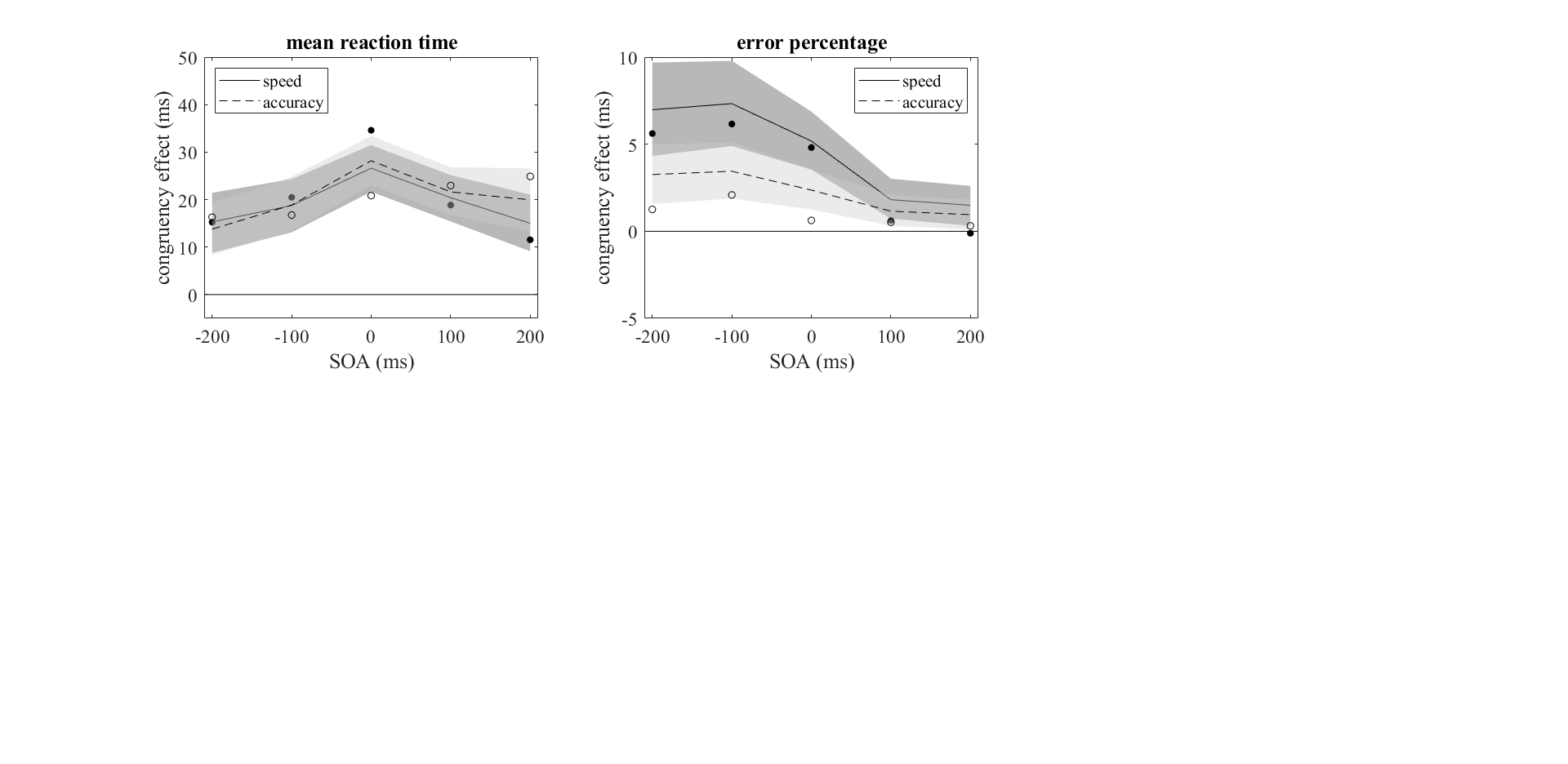

Supplement: Supplement. — The supplement describes a model-based analysis of the results reported in the main article. [file joc-6-1-318-s1.zip › s1-joc-318_wuhr/Fig. S6.tif]
